# Metallic Nanowires Self-Assembled in Quasi-Circular Nanomolds Templated by DNA Origami

**DOI:** 10.3390/ijms241713549

**Published:** 2023-08-31

**Authors:** David Daniel Ruiz Arce, Shima Jazavandi Ghamsari, Artur Erbe, Enrique C. Samano

**Affiliations:** 1 Centro de Nanociencias y Nanotecnología, UNAM, Ensenada 22860, Mexico; ddruiza@ens.cnyn.unam.mx; 2 Helmholtz-Zentrum Dresden—Rossendorf, 01328 Dresden, Germany; s.jazavandi-ghamsari@hzdr.de; 3 Cluster of Excellence Center for Advancing Electronics Dresden (cfaed), TU Dresden, 01187 Dresden, Germany

**Keywords:** DNA nanotechnology, nanoelectronics, self-assembly, nanomaterials

## Abstract

The self-assembly of conducting nanostructures is currently being investigated intensively in order to evaluate the feasibility of creating novel nanoelectronic devices and circuits using such pathways. In particular, methods based on so-called DNA Origami nanostructures have shown great potential in the formation of metallic nanowires. The main challenge of this method is the reproducible generation of very well-connected metallic nanostructures, which may be used as interconnects in future devices. Here, we use a novel design of nanowires with a quasi-circular cross-section as opposed to rectangular or uncontrolled cross-sections in earlier studies. We find indications that the reliability of the fabrication scheme is enhanced and the overall resistance of the wires is comparable to metallic nanostructures generated by electrochemistry or top-down methods. In addition, we observe that some of the nanowires are annealed when passing a current through them, which leads to a clear enhancement for the conductance. We envision that these nanowires provide further steps towards the successful generation of nanoelectronics using self-assembly.

## 1. Introduction

The enormous advances in the semiconductor industry have changed how people can interact with each other, receive healthcare, and work more efficiently, just to mention a few examples [1]. The production of smaller and cheaper electronic devices is foremost for future developments in electronics. The control of feature sizes below 10 nm in the fabrication of next-generation devices for sensing, computing, data storage, and communication systems is essential for reaching this target [2]. Developing novel, revolutionary materials and nano-manufacturing methods are required to achieve it. However, there is no doubt that the scientific and technological hurdles look almost insurmountable. An alternative way to overcome such hurdles is combining bottom-up and top-down techniques, which could be a roadmap to move into the right direction [3].

The convergence of recent advances in the DNA-based self-assembly approach and surface modification chemistry promises to bridge bottom-up and top-down schemes, enabling efficient construction of a new generation of devices [4,5,6]. Although non-origami DNA nanostructures have been reported [7], the cost-effective DNA Origami technique allows for the generation of 2D and 3D structures and for the controlled positioning of metal nanoparticles (NPs) [8]. Electronic devices consist of materials having a wide range of electrical conductivity values. Therefore, multilayer devices are fabricated using the deposition of insulating, semiconducting, and metallic overlayers, followed by patterning with photolithography and reactive ion etching in an ultra-clean room. On the contrary, a major breakthrough would be using simple, not-so-immaculate, but effective processes occurring in living species, namely, the self-assembly of organic molecules and macromolecules, as a first stage for manufacturing semiconductor devices [9].

A general scheme is devised in this work to fabricate gold nanowires (Au NWs) using custom-tailored 3D modules at the nanoscale. The modules are synthesized through the DNA Origami technique by hybridizing a scaffold and many short linear DNA single strands, ssDNA, complementary to the scaffold sequence [10,11].

**Figure 1:**
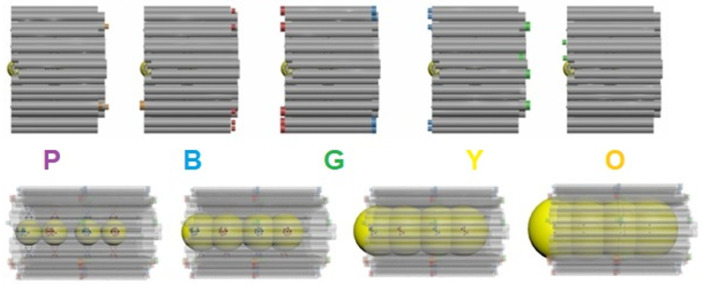
Coupling of five modules **P—B—G—Y—O** using extended staples to create the basic DNA nanostructure (**top**). Evolution of the metalization process by electroless metal deposition on Au NPs to achieve a gold nanowire, the DNA nanostructure is being used as a mold (**bottom**).

The size and shape of the fundamental module, a hexagonal cross-section cylinder, is methodically conceived using a freeware named caDNAno. Each NW is based on a basic nanostructure, formed by assembling five modules with a biunique coupling using short extensions of complementary ssDNA of face-to-face neighboring modules, as observed at the top of Figure 1. To discriminate them, the five modules were labeled with different colors: purple—P, blue—B, green—G, yellow—Y, and orange—O. At each module, two Au NPs are previously attached at specific binding sites [12]. This approach allows for the casting of continuous Au NWs due to the precise positioning and controlled growth of the Au NPs by chemical reduction within the DNA basic nanostructure [13]. This two-step process is shown at the bottom of Figure 1. The shape of the seamless nanostructure reduces stress when the space inside the nanostructure is filled during this metalization procedure, and the resistivity of the resulting NWs is expected to decrease. The present study focuses on the feasibility of manufacturing high-quality metallic wires produced by controlling and confining the metal growth within three-dimensional mold nanostructures in contrast to our previous work using two-dimensional DNA Origami [14]. This demonstration of a first step towards nanofabrication using self-assembly will allow for the future development of nanoelectronic components once the generation of other metallic and semiconducting materials has been achieved using similar strategies. On top of that, self-recognition schemes, as presented here, can be used for biosensing [15], thus opening the way for further applications.

Au NWs are mainly used as microchip interconnects [16]. The NWs must be uniform and continuous to minimize their resistivity for this application. The initial stages of nucleation on Au NPs fixed to the DNA nanostructure and growth process are critical to satisfying these requirements. The first studies about the synthesis of DNA-templated NWs involved the coalescence of gold along DNA strands, either single or double [17]. The structure of the obtained NWs was a beads-along-a string morphology, and a major tour de force was the transformation from this framework to a neat metallic object having uniform geometry. The growth mechanism involves nucleation at binding sites on the DNA followed by the growth of spherical particles. Eventually, under favorable conditions, the final morphology of the overlaid metal is determined by two competitive processes: surface tension and adherence to the DNA template [17]. Although this effect has been observed in the fabrication of DNA-templated NWs based on the coalescence of gold around DNA linear strands immobilized on mica, their inferences can be extended if the templates are 3D DNA Origami nanostructures instead. Future studies will concentrate on improving the mechanical stability and cohesion of the actual DNA basic nanostructure, which serves as a template for the efficient assembly of the Au NPs, to enhance the electric conductance of the resulting NWs.

Gel electrophoresis, Atomic Force Microscopy (AFM), Transmission electron microscopy (TEM), and scanning electron microscopy (SEM) analytical techniques characterize the nanostructures. Randomly distributed Au NWs with a size below 200 nm were laid down on SiO_x_/Si substrates and contacted with gold electrodes by electron beam lithography (EBL): A study of charge transport at room temperature of these wires shows ohmic behavior of the contacts. In addition, a reduction of the electrical resistance resembling current-induced annealing at atmospheric pressure is found. The conductance of the NWs shown here based on DNA nanostructures can possibly be explained by the so-called wind forces. The manufacturing of these Au NWs might provide a proof-of-concept for the feasibility of top-down nanofabrication combined with self-assembling systems, a long sought goal.

## 2. Results and Discussions

### 2.1. Visualization of the 3D Nanostructures

**Figure 2:**
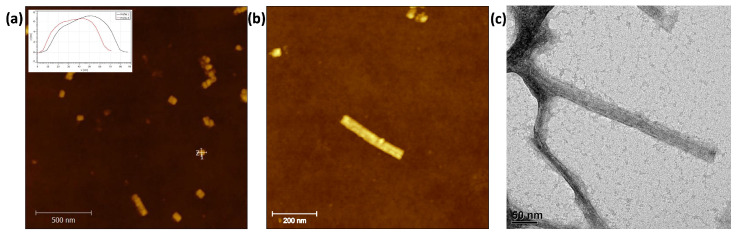
(**a**) AFM image, 2.5 μm × 2.5 μm image of the “blue” module deposited on SiO${}_{x}$/Si. (**b**) AFM image, 0.6 μm × 0.6 μm image, of the basic nanostructure on SiO${}_{x}$/Si. (**c**) TEM micrograph of the DNA basic nanostructure used as the mold.

**Figure 3:**
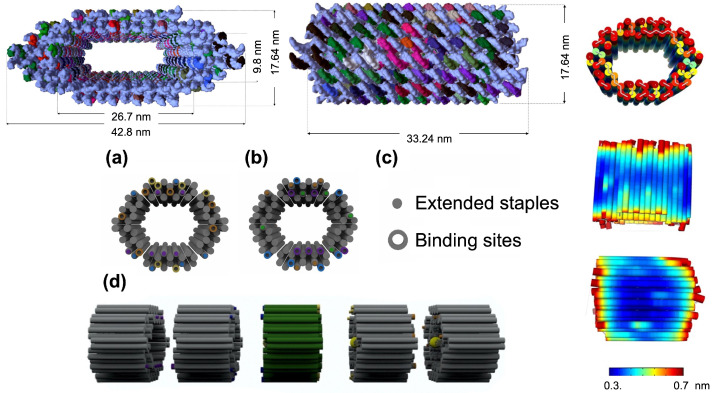
(**Upper left**) Molecular representation of the fundamental module as accomplished by means of Molecular Viewer from Autodesk. (**Upper right**) CanDo simulation for studying the structural stability of the primary module. (**Lower**) Schematics showing extended staples and binding sites for the unique coupling of the modules. (**a**) Back and (**b**) front view of a module. (**c**) The symbols are solid circles for extended staples and hollow ones for binding sites. (**d**) Assembly of the basic DNA nanostructure.

After annealing the scaffold with the staples and purifying the resulting modules, the structures were investigated using SEM, TEM, and AFM. The imaging of samples was first carried out utilizing tapping mode AFM in air. Figure 2a) shows a characteristic AFM image for the “blue” module. Most of the structures in the figure correspond to well-shaped single loose “blue” modules. However, the yield is low, with a few secondary structures, two to four modules overlapped by DNA-base stacking [18], already predicted by gel electrophoresis (not shown here). A cross-section analysis was also conducted, resulting in 36 nm and 32 nm for length and width, respectively. A similar visualization and cross-section analysis in AFM were also made for the other four modules, see Supplementary Information E Figures S8–S12. Table 1 summarizes the most relevant dimensions. These values agree with those predicted by the caDNAno design: length ~33 nm and width ~30 nm. The width measurement is an approximation because the module has a hexagonal cross-section. According to the Molecular Viewer model, based on the caDNAno design, the minimum and maximum width values are 17.6 nm and 42.8 nm, respectively. The physical contact between the specimen and tip apex when rastering during surface imaging is one of the characteristics of AFM as a topographic tool for sample analysis. The width of the hollow 3D module is distorted when the AFM tip scans a soft sample. Even in the tapping mode, the tip flattens the DNA module due to the normal stress on it; see the height values in the inserted cross-section in Figure 2a. This is why it is reasonable to compare the measured value with the width average value of the molecular model, ~30 nm, in Figure 3 upper left.

All modules were bound together in a subsequent hybridization step to shape the basic nanostructure. Figure 2b shows a typical AFM image of an assembled DNA basic nanostructure with a ~190 nm length and ~30 nm width. As expected, no gaps were observed in the coupling of adjacent modules. The continuity of the mold, DNA basic nanostructure, was verified by TEM as well, a visualization technique with a high resolution. Figure 2c shows the image of two joined seamless molds with a 28 nm width.

**Figure 4:**
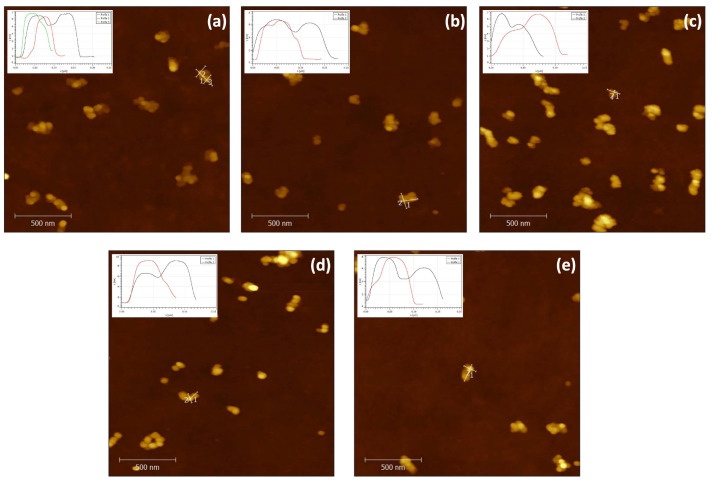
AFM micrographs of Au NP–module bioconjugates for the **P**, **B**, **G**, **Y**, and **O** modules. The scale bar is 500 nm.

As already mentioned, one of the advantages of the DNA Origami approach is locating and fixing inorganic material with a precision at the nanoscale. After functionalizing short ssDNA with Au NPs, two Au NPs with a 5 nm size and a separation of ∼16 nm were inserted within each module, one in the middle and the other at one of the edges. By this means, Au NP–DNA Origami bioconjugates were generated by annealing the purified functionalized Au NPs, previously re-diluted in the proper buffer, with each module. The Au NPs insertion in each module was subsequently verified by AFM. According to the cross-section analysis in the AFM image insets in Figure 4, Au NPs were found inside some Au NP–module bioconjugates, as expected, but the yield is low. The measured distance between Au NPs is unreliable, as inferred from the images in Figure 4, due to the fact that the Au NPs are in the interior of the module and the functionalized ssDNA–Au NPs are flexible. The main function of the Au NPs is to become nucleation centers when gold coalesces on their surfaces during the metalization process.

**Figure 5:**
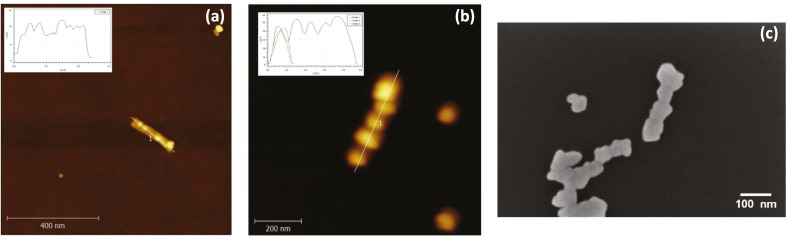
(**a**) AFM image of the basic nanostructure functionalized with Au NPs on SiO_x_/Si. Seven NPs can be clearly identified, where one of the NPs is just off the straight line where the cross-section was made. AFM (**b**) and SEM (**c**) images of a Au NW on a SiO_x_/Si substrate before being electrically contacted by EBL.

After verifying the feasibility of Au NP–module bioconjugates, shown in Figure 4, and the synthesis of continuous basic nanostructures without Au NP, shown in Figure 2b, the mold formation for the Au NWs is followed. The Au NP–basic nanostructure bioconjugate, or mold, was shaped by the biunique assembly of the five Au NP–module bioconjugates. An AFM image of the basic nanostructure functionalized with Au NPs within is shown in Figure 5a. It is inferred from the cross-section analysis that the nanostructure has a length of ~200 nm and width of 35 nm with 7 Au NPs inside. It is worth mentioning that 8 Au NPs, instead of 10 as designed, was the highest number of NPs which could be found in all images. The yield of NP attachment was thus observed to be low as well. This was probably due to steric effects within the nanostructure. Even though the inner cross-section for the basic nanostructure is minute, the literature reports the synthesis and characterization of Au NWs as having a comparable size to the one reported here [19]. Since the gaps between the NPs are filled by the subsequent metalization step, we can continue to form NWs even though some particles are missing inside the structures.

Finally, elongated Au NWs were created by the metalization of the Au NP–basic nanostructure bioconjugates and laid down on a SiO_x_/Si substrate. The main purpose of the bioconjugate nanostructures is to be utilized as molds when the Au NWs are cast, which is the main object in this part of the fabrication process. Figure 5b shows the AFM cross-section analysis of a typical Au NW having dimensions of 360 nm and 56 nm in length and width, respectively. The structure of the extended Au NW is grainy rather than smooth, although it looks continuous. These dimensions and morphology of a similar NW were verified by SEM, Figure 5c, resulting in similar values for length and width. This type of structural appearance has been already observed in previous publications [20,21]. A successful metalization increases the chance to synthesize continuous and “almost-cylindrical” Au NWs due to an efficient reduction of gold on the Au NPs to enlarge their size, join them, and pack the available space inside the DNA container. The electron transport study in these Au NWs was then performed.

### 2.2. Electrical Characterization

**Figure 6:**
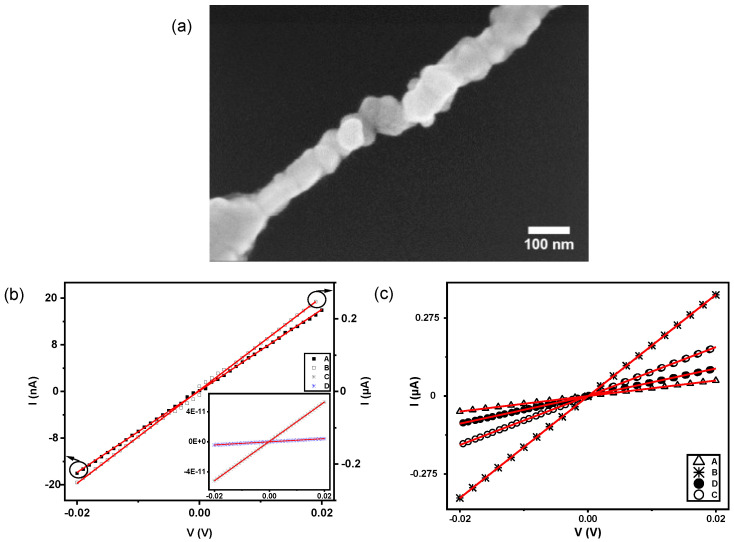
(**a**) A Au NW with two thermal contacts wired to the pads in the chip. (**b**) *I*-*V* measurements at room temperature (RT) and ambient pressure of four NWs using the “ambient setup”. The left and right y-axis refers to the samples labeled as A and B, respectively. The inset corresponds to the *I*-*V* curves of the other two samples, labeled as C and D. (**c**) *I*-*V* measurements at room temperature (RT) in a system at a pressure of 1 × 10^−6^ mbar of the same devices displayed in (**b**) but using the “vacuum setup”.

After fabricating and successfully metalizing the NWs on a SiO_x_/Si substrate, EBL was utilized to contact the NWs using alignment markers. Nine NWs were connected with two metallic contacts for defining source and drain electrodes in the electrical transport measurements; one example is shown in Figure 6a.

Initial measurements at room temperature and atmospheric pressure revealed a large variety of resistance values; characteristic *I*-*V* curves are shown in Figure 6b. Due to the limited voltage range, all curves are linear; therefore, a resistance value can be deduced for all NWs from the slope of each *I*-*V*-curve. One of the wires was conductive with a low resistance value of 78 Ω, while the other three have much higher resistances ranging from 1.5 × 10^6^ Ω to 5 × 10^9^ Ω, as shown in Table 2. The remaining five NWs were either short-circuited due to failure in the EBL process or no significant current was measured above noise level. SEM micrographs of the interconnects at every NW are shown in Supplementary Information F (Figure S13A,B). The high resistance values could be explained by the imperfect connection of the NWs to the Au-contacts, or because they might not be homogeneous and continuous, e.g., the existence of a gap, or gaps, somewhere along the wire. The most plausible argument of these two will be examined below.

The electrical measurements were reproduced in a different setup, termed the “vacuum setup” in the Methods section. In particular, Figure 6c shows the *I*-*V* curves of the same four previous NWs at room temperature from Figure 6b recorded using the “vacuum setup”. The resistances were determined again from the linear fit to the measured *I*-*V* curves. Interestingly, the resistances were in the 56 Ω to 370 Ω range, as shown in Table 2. We now found a significant reduction in the resistance values for all wires, which were not damaged during the procedure (some of the wires were destroyed by electric shock). Even the NWs which showed tiny currents in the initial measurements were conductive in the same range as the other wires in the subsequent measurements. This decrease in resistance may be explained either by an alteration in contacts or by a modification of the morphology during the initial measurements. The resistance decrease is most prominent for the initially highly resistive wires, while it is only weak for the initially conductive wires because they are already in a low resistance state. To explain this extraordinary behavior in electron transport in metallic NWs, we compare our results to studies on similar systems in recent literature.

The use of 2D and 3D DNA Origami nanostructures to be harnessed as templates for fabricating Au NWs with dimensions and morphology determined by their geometry and shape has been reported recently [22,23]. Woolley et al. realized electric measurements at ambient conditions of C-shaped Au NWs, 130 nm long, laid down on 70 nm × 90 nm planar DNA Origami. They found resistance values in the range from 5.6 × 10^3^ Ω to 11.7 × 10^6^ Ω using four-probe contacts, which were defined by EBID; no post-deposition annealing was conducted on the NWs. [24]. Erbe et al. also used a self-assembled rectangular DNA Origami 90 nm × 70 nm) as a template to fabricate C-shape Au NWs with an overall length of 150 nm. The attachment probability of the Au NPs constituting the NWs was optimized for the desired patterns. To complement the previous studies, the electrical properties of these NWs were tested at temperatures ranging from 4.2 K to RT (293 K). Two C-shape NWs showed ohmic behavior at RT, and the resistances were found to be 2.3 × 10^6^ Ω and 1.4 × 10^9^ Ω. Despite their reduced dimensions and curved shape, these wires are as good in electrical quality as those previously manufactured, namely, longer straight wires [14]. Based on previous work, Woolley et al. studied the impact of low-temperature annealing on the morphology and conductance of DNA-patterned straight NWs at ambient conditions [25]. They found that the resistance of the wires decreased after being PMMA coated and annealed at 200 ${}^{\circ }C$ with four-point electrical measurements using EBID-defined contacts. The average NW resistance changed from 6.28 × 10^6^ Ω (pre-annealing) to 1.90 × 10^5^ Ω (post-annealing). For nanosized Au conductors, Rayleigh instabilities can cause the nanostructures to break up into small droplets even well below the melting temperature [26]. The authors claim that the diameter of the annealed structure is constrained by the PMMA coating, inhibiting the Rayleigh instability to some degree and imposing a mechanical constraint on the movement of gold. The presence of the thermally conductive EBID metal contacts, tungsten in this case, is not expected to influence the annealing of the Au NWs because both are expected to be at the substrate temperature [25].

The 3D nanostructures with an inner hollow have shown so far the best results in terms of electric conductance. Seidel et al. synthesized DNA nanostructures with a square cross-section, 40 nm long, 23 nm and 14 nm for outer and inner sides, respectively, using a modified version of the ssDNA M13mp18 genome having 7560 nt [27]. Later, they utilized this approach to couple DNA Origami mold monomers into a long linear structure to assemble conductive Au NWs [19]. The total length of each Au NW varied from 110 nm to 1000 nm with a diameter of 32(3) nm. Each mold monomer had a square cross-section with identical dimensions as in a previous report [27] but now had a 5 nm DNA-functionalized Au NP each to be used as a seed. The resistance values for approximately ten 200 nm-long NWs were widely spread from 90 Ω to 9 × 10^9^ Ω [19]. The inhomogeneous growth of the Au NPs might be responsible for the discontinuity of the NWs and, therefore, the increase in resistance. They concluded that the charge transport of electrons in the DNA Origami template Au NWs was dominated by a single non-metallic interparticle interface that needs to be overcome by hopping at higher temperatures. An alternative method is using templates based on 3D DNA Origami nanostructures with a cross-section as close as possible to a circular shape to reduce non-metallic gaps at the interfaces. The results shown here in Figure 6 and Table 2 indicate how the conductance improves in Au NWs templated by 3D Origami if the cross-section is almost-circular, actually hexagonal in the design, possibly due to the reduction of internal strain of the metal confined to the mold during growth. Actually, the resistance values found here, see Table 2, are in the same order of magnitude to those reported in the literature, see Table 3 in [23], for similar 3D nanostructures.

An argument of plausibility will now be given to elucidate the huge variation in resistance of three out of four NWs after using two setups in different environments, compare Figure 6b,c. A simple explanation of these experimental results is provided in the spirit of Occam’s razor. The high resistance values found in the devices utilizing the “ambient setup” could be explained by the possible existence of voids or vacancies within the NWs; i.e., they were probably neither completely continuous nor homogeneous after the metalization process. When passing current through such an NW, two forces act on the ions constituting the lattice of the wires: the direct electrostatic force on the electrons, and a possible contribution to the annealing of the wire by the so-called wind force. The wind force arises from the momentum transfer of electrons to metal ions, impurities, grain boundaries, vacancies, dislocations, and even phonon vibrations of ions from their equilibrium positions via scattering events when an electric field is applied [28]. These scattering processes thermally activate the migration of atoms by directionally decreasing their activation energies, $E_{a}$, and consequently result in a directed diffusion. The resulting material migration occurs in the direction of the electron flow. At low voltages, in a range from −20 mV to 20 mV, atoms or defects with the smallest activation energy barrier will become mobile in the contact region, move out of the contact due to diffusion, and redeposit in cooler regions [29]. Atoms located at dislocations, grain boundaries, surfaces, and vacancies have lower binding energies and, as a consequence, lower $E_{a}$ values. An $E_{a}$ value of 0.8 eV has been measured, even at low current densities, for passivated Au interconnects in which grain boundary diffusion acts as the dominant mechanism [30]. It should be noted that the electric fields arising in the structures are much larger than the fields required to induce the electrophoretic motion of isolated Au NPs in ds-DNA [31]. This further indicates that the removal of the DNA mold and the formation of continuous wires has been completely achieved in our NWs.

Due to difference in electrothermal properties, grain structure, the contribution of different atomic diffusion pathways, and the development of mechanical stresses, the wind-force-driven annealing in NWs differs from micro-scale interconnects in integrated circuits [32]. This process in NWs starts at the location of the highest divergence of atomic flux, which depends on the spatial profile of temperature, current density, and scattering cross-section. As mass is ejected from that location, the NWs narrow down over a length of approximately 10 nm [33]. Thinner and narrower Au wires are known to generate lower heat when the electron–phonon scattering length is on the order of 170 nm [34]. This whole process changes the electric resistance due to this force exerted on each metal ion core. This simple mechanism might fill the existing vacancies at the end of the experimental run in the “ambient setup”, turning the Au NWs more conductive than before. As plating is typically used to produce conductive NWs, in general, annealing of plated NWs is highly relevant to the fabrication processes for NW electronics.

## 3. Materials and Methods

### 3.1. Module Design

The elongated Au NWs are cast using a 3D nanomold having a DNA module as the primary unit. This structure should withstand mechanical stress and keep its configuration during casting. To achieve these requirements, the modules used in this work are based on a variation of that created by the Peng Yin group [35], a rigid hollow 3D nanostructure synthesized by means of the DNA Origami method [10,36]. The fundamental module designed by caDNAno corresponds to a hollow tubule having a hexagonal cross-section formed by 80 helices. The module is attained by hybridization of a scaffold and several staples mixed in a buffer at the proper conditions. The Molecular Viewer software was used to obtain a quasi-realistic outline to scale by following bonds and bond angles based on the files provided by caDNAno [37], as shown in Figure 3. The design of the five modules shaping the nanomold can be simplified if the scaffold is precisely the same as the one used by the Peng Yin group, but the length and sequence of staples near the ends of the modules have been modified. By this means, the coupling of modules would be seamless because the extended staples simply bind with the complementary ones of those located close-by; no voids are left around the junction of modules (see Table S1 and the paragraph below the caption in Supplementary Information A for further information). This “peg-notch” assembly keeps the structural stability of the 3D nanomold.

Each module was ~33 nm long with ~30 nm and ~18 nm average outer and inner diameters, respectively, as observed in Figure 3 upper left. The mechanical and structural stability of each module was then studied using the CanDo computer code [38] based on the files generated by caDNAno. Flexibility maps of the DNA basic module assembly were determined and the thermally induced fluctuations were found to be in the 0.3 nm to 0.7 nm range, as shown in Figure 3 upper right. Similar results in CanDo were obtained for all five modules. The careful iterative use of caDNAno and CanDo design and analysis increases the chances of successfully synthesizing a DNA nanostructure. This whole method assures a high level of confidence in the modules’ fabrication.

### 3.2. Basic Nanostructure Design (Five-Module Assembly)

The NWs’ length is determined by an integer number of nanomolds, similar to links in a catena. For the sake of simplicity, the modules have been tagged with “colors” to distinguish them: P—B—G—Y—O. The main modification in the design of each module is harnessing the exceptional Watson–Crick base pairing between purines and pyrimidines, having the possibility to join adjacent modules in a particular manner. This is accomplished by extending certain staples, which have a minimum length of 8 nt, away from a module to be complementary to the scaffold of a neighboring module. As shown in Figure 3 lower, the front of the purple module (PF) links to the back of the blue one (BB); the front of the blue module (BF) connects to the back of the green one (GB); the front of the green module (GF) couples with the back of the yellow one (YB); and finally, the front of the yellow module (YF) joins to the back of the orange one (OB). In this case, the basic module [35] is the green one. The main goal of the nanostructure design is to prevent a union between undesired modules and avoid gaps around the faces of consecutive modules. This unique coupling between different modules allows for accomplishing a final basic nanostructure with controlled cross-section and length, resulting in a gold NW that is structurally stable and stiff.

### 3.3. Au NP–DNA Origami Bioconjugate Design

**Figure 7:**
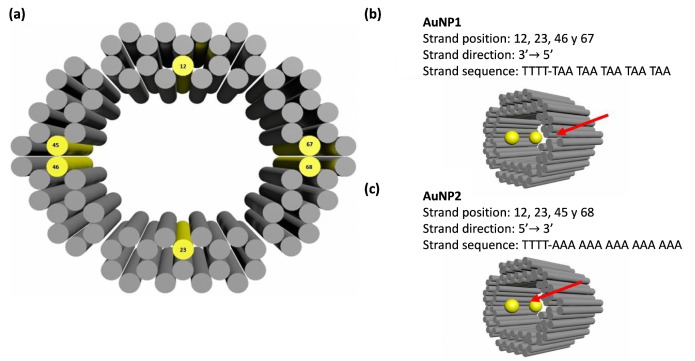
Au NPs location within the DNA Origami module with a “cylindrical” shape. (**a**) Selected helices (solid yellow circles) for extending some staples for attaching Au NPs. (**b**,**c**) Positions in the module and sequences of extended staples for capturing Au NP1 and Au NP2, respectively.

After functionalizing Au NPs with the suitable single strands, two NPs, 5 nm in size each, were positioned at the front and middle of each module, separated ~16 nm from center to center. This procedure was carried out before coupling the modules. Each Au NP was attached by four binding sites to the module. The sequence of the capturing strands for the Au NPs used here was devised by the Tim Liedl group in such a way that they point to opposite directions [39], i.e., the extended staples in each module for capturing Au NP1 and Au NP2 are directed along the $3^{\prime }\to 5^{\prime }$ and the $5^{\prime }\to 3^{\prime }$ directions, respectively, as displayed in Figure 7. The design aims to increase the binding probability for each NP. Similarly, the sequence of the functionalized Au NPs is complementary to those of the extended staples, not shown here. To ensure that each extended staple is pointing towards the module’s interior, the staples were chosen using the Maya AutoDesk software to consider the intrinsic helicity of DNA [40].

### 3.4. Synthesis of Modules

The primary module is annealed by hybridization of a scaffold and several staples (Integrated DNA Technologies, Inc., Coralville, IA, USA) mixed in a buffer at the proper concentration and pH. The scaffold is a modification of the M13mp18 plasmid, 7249 nt, with a total length of 7249 + 311 nt, also termed as p7560 (Tilibit Nanosystem, Inc., Munchen, Germany). After optimizing the design for all modules and extended staples, the original plates and tubes containing the staples and scaffold were centrifuged to settle their content. Then, their actual concentrations were verified using a NanoDrop 2000 Spectrometer (Thermo Scientific, Inc., Waltham, MA, USA). These values resulted to be almost identical to the nominal concentrations for the p7560 single-strand scaffold, 100 nM, and staples, 200 μM. As a strategy for making easier and more efficient the synthesis process for the five modules, the standard and extended staples were split into “pools”, namely, vials containing a small amount of solute in a standing buffer. In our case, 13 different “pools” were prepared to shape different regions of each module and capture Au NPs at the desired locations (see Supplementary Information A and B for further information).

All pools were prepared by pipetting the suitable staples, blending them, and diluting them to a final concentration of 0.5 μM for each. A description of the necessary volumes of scaffold, buffer, and variety of pools for annealing all five modules is described in Supplementary Information B (Tables S2–S6). The mixture for each module was pipetted in a PCR microtube and annealed in a programmable thermal cycler; the temperature was raised to 80 ${}^{\circ }C$ and kept constant for 5 min, then the temperature was gradually decreased in steps: from 79 ${}^{\circ }C$ to 56 ${}^{\circ }C$ during 348 min, from 55 ${}^{\circ }C$ to 46 ${}^{\circ }C$ during 225 min, and from 45 ${}^{\circ }C$ to room temperature (25 ${}^{\circ }C$) during 302 min. Afterward, the prepared modules were filtered by performing four buffer exchanges in a 1 × TAE/ 12.5 mM Mg${}^{2+}$ buffer (40 mM Tris-acetate, 1 mM EDTA, pH 8.2) with a Microcon DNA Fast Flow centrifugal filter (100 kDa MWCO, Millipore, Burlington, MA, USA) to remove the non-hybridized extra staples. The final concentration of each module was measured using a NanoDrop and resulted to be 10 nM.

### 3.5. Visualization of Modules and Nanomolds

Gel electrophoresis was performed to reveal the formation of each module and extract the modules for further preparation. An image of one of the resulting gels is shown in Supplementary Information C (Figure S1). The resulting nanostructures were analyzed by TEM. A Lacey carbon grid (Ted Pella, Inc., Redding, CA, USA) was employed to deposit 2.5 μL of a sample after annealing. The sample was deposited and adhered to the grid’s network after waiting for 3 min, and then 2.5 μL of uranyl acetate at 2% was added for staining and incubated for 5 min. Afterward, a JEOL JEM 2000 FX TEM was used for imaging. Apart from TEM, all visualization and electrical characterization techniques require an insulating substrate. The modules, basic nanostructure, and Au NWs were deposited on 1 cm × 1 cm pieces of p-Si (100) wafer with a 300 nm SiO${}_{2}$ electrical insulation layer, named SiO${}_{x}$/Si substrate. Before dropping the DNA Origami, the wafer pieces were washed with ethanol and dried using a N_2_ flow. They were pre-treated in an O_2_ plasma (PICO, Diener Electronic-Plasma Surface Technology) at 7sccm O${}_{2}$, power of 240 W for 5 min to render their surfaces hydrophilic. At that moment, each sample holder was rinsed with ethanol (20 s) and DI water (20 s) and dried in a N${}_{2}$ stream. A volume of 20 μL of the sample was immediately dropped on the substrate surface at a final concentration of 1 nM, or 0.5 nM if 10 × TAE Mg${}^{2+}$ buffer was added, and incubated for 1 h. Afterward, the substrate was washed using HPLC-grade water and carefully dried in a N${}_{2}$ stream. AFM and SEM then studied the morphology of all samples. The DNA assemblies were characterized by AFM operating in tapping mode using a Bruker MultiMode 3 and Bruker MultiMode 8 Scanning Probe Microscopes and aluminum reflex coated tips (Tap150Al-G from Nanoandmore, force constant 5 N/m^−1^, tip radius < 10 nm) for obtaining high-resolution images. The topographic AFM images were analyzed using the Gwyddion software (http://gwyddion.net/) and the one provided with the Bruker instruments (Billerica, MA, USA). To study the proper concentration of a divalent cation for stabilizing the DNA Origami modules and diminishing aggregation when specimens were laid down on the SiO${}_{x}$/Si substrates, samples containing 125 mM, 200 mM and 350 mM Mg${}^{2+}$ diluted into 10 × TAE (40 mM Tris-acetate y 1 mM EDTA, pH 8.3) were prepared, and module deformation and aggregation was minimized when pipetted at 200 mM, see Supplementary Information D (Figures S2–S7). It was found that adding Mg${}^{2+}$ at 200 mM into the buffer was the proper amount. After proving that the nanomold can be constructed and analyzed by TEM and AFM, the casting of the NWs was carried out by a two-step process. Firstly, two Au NPs were placed inside each module. Secondly, an electroless chemical solution was used to reduce gold on the Au NPs surface to grow and merge them, filling the existing space within the stiff DNA mold.

### 3.6. Annealing of Au NP–Origami Bioconjugates

The Au NPs (Ted Pella, Inc.), 5 nm in size and nominal concentration of 5 × 10^13^ particles/mL, were conjugated to the specific oligonucleotide chains, complementary to those shown in Figure 7. The sequences of the capture strands for Au NP1 and Au NP2 are ATT ATT ATT ATT ATT TTTT-SH and TTT TTT TTT TTT TTT TTTT-SH and directed along the $5^{\prime }\to 3^{\prime }$ and the $3^{\prime }\to 5^{\prime }$ directions, respectively. The capture single strands had a thiol (SH) modification on either the $5^{\prime }$ end or $3^{\prime }$ end for binding to a Au NP, followed by a 4-base spacer (TTTT) for increased flexibility. The “salt aging” method was used to neutralize the inherent charge in Au NPs and thiolated single strands; both have a negative charge and still keep the colloidal stability of Au NPs. This method was followed for both Au NP1 and Au NP2 and has been previously reported [20]. Later, the disulfide–DNA sequence was added and mixed to the solution at a ratio of 1:5 Au NPs: ssDNA, left in a TekTrator^®^ V type shaker at 120 rpm, and allowed to incubate for ≈48 h. After incubation, the Au NPs were backfilled with thiolated T5 sequences at a ratio of 1:60 to prevent Au NPs aggregation in the presence of high Mg^2+^ concentration buffers and left incubating for another 24 h. Excess DNA single strands were removed from the Au NPs–ssDNA conjugates by running the bioconjugate solutions for each Au NP on two 3% agarose gels (1 × TAE Mg^2+^, 12.5 mM) for 15 min at 100 V and 4 ${}^{\circ }C$. The band containing Au NPs–ssDNA with the highest concentration (the most intense) was sliced and extracted from the gel, then finely chopped and centrifuged using a Freeze’N Squeeze kit (Bio-Rad Labs, Tokyo, Japan). Next, 1 × TAE buffer was added to the microtube containing the Au NP1 and Au NP2 bioconjugates until it reached the final concentration of 200 nM for each, measured using a NanoDrop. The final solvent was assumed to be 1 × TAE buffer with residues from the Freeze´N Squeeze product and the agarose gel. Now the Au NP–Module bioconjugates are ready to be hybridized. The Au NPs were attached by four binding sites, being the functionalized Au NPs complementary to those of the capturing strands in each module. The protocol for this part is almost identical to that developed by our group and found elsewhere [19]. A ratio of 6:1 of Au NP–ssDNA: Module was prepared to increase the likelihood of success. The original concentrations of Au NPs–ssDNA conjugates and each module resulted in 200 nM and 10 nM, respectively. Therefore, the following volumes: 0.6 μL of Au NP1–ssDNA, 0.6 μL of Au NP2–ssDNA, 2 μL of the module, and 16.8 μL of 1 × TAE were pipetted in five microtubes for each module. This means that the final concentrations of 1 nM for DNA Origami and 6 nM of Au NPs would be obtained in a volume of 20 μL in each vial. To ease and assure that the Au NP:ssDNA conjugates were transferred from the solution to the interior of each module, a sideral shaker with a heater (ThermoMixer Eppendorf) was used. The five vials containing each sample were secured on the shaker, warmed to 40 ${}^{\circ }C$, then gradually cooled to 23 ${}^{\circ }C$ (room temperature) for 5 h at a frequency of 300 rpm. Later, the samples were imaged by AFM in the tapping mode in air. As mentioned, the Au NPs–Mold bioconjugates were obtained by hybridizing the five Au NPs–modules bioconjugates. Microtubes containing the same stoichiometry and volume, 5 μL, for each module bioconjugate, were pipetted and mixed in a buffer containing 5 mM Tris-HCl, 1 mM EDTA, 11 mM mM MgCl${}_{2}$, pH 8.0, with 350 mM NaCl and incubated overnight. Thus, the final basic nanostructure, or mold, was formed and ready to be metalized.

### 3.7. Metalization of Au NP–Origami Bioconjugates

Subsequently, Au NPs within the mold were enhanced until they merged to shape quasi-cylindrical Au NWs. The growth of the Au NPs was controlled using the GoldEnhance^TM^‚ EM Plus (Nanoprobes, Inc., Yaphank, NY, USA) metalization kit having four homogeneous mixtures. Same volumes, 10 μL, were taken from each solution, 1:1 ratio, and mixed with 40 μL of 1 × TAE buffer, 1:2 ratio, with 20 mM MgCl${}_{2}$. This gold plating mixture, 80 μL, was separated into four equal volumes and dropped onto the Au NP–Origami conjugates, which were laid down on a clean SiO${}_{x}$/Si substrate at equal intervals for a total of 10 min using a humidity chamber. Afterward, HPLC water was thoroughly sprayed onto the sample to inhibit the metalization procedure, and finally, N${}_{2}$ was carefully blown to dry it. The Au NWs were ready to be contacted by EBL.

### 3.8. Fabrication of Contacts and Electric Characterization

A RAITH e-line Plus system was used to analyze the samples’ morphology by SEM, fabricate the electric pads, and contact the NWs using alignment markers by lithography. EBL (e-line Plus) was used to manufacture the electrical contact pads and markers on p-Si (100) with a ≈280 nm thick SiO_x_ electrical insulation layer, previously plated with DNA-Origami-based gold wires. This random arrangement of NWs was firstly treated in an O_2_ plasma (PICO, Diener Electronic-Plasma Surface Technology, Ebhausen, Germany) at a flow rate of 5 sccm, input power of 240 W for 30 min to minimize any amount of organic residues on the sample. Then, the ZEP 520A electron beam resist was spin-coated and baked for 10 min at 150 ${}^{\circ }C$. The resist was exposed to a 10 kV acceleration voltage with a 30 μm aperture size, resulting in a beam current of ≈200 pA. To remove the resist in the exposed structures on top of the NWs, the sample was developed for 90 s in N-amyl acetate and subsequently immersed for 30 s in isopropanol (IPA) to stop the development. An e-beam evaporator Creavac CREAMET 600 (CREAVAC) was used to evaporate a 5 nm thick titanium adhesion layer initially and then a 90 nm gold deposit with 2 Å s^−1^ and 5 Å s^−1^ rates, respectively. The lithography was completed with a lift-off process that included 90 s in N, N-dimethylacetamide (ZDMAC), 30 s in the stopper (Isopropyl Alcohol), and dried by a N_2_ flow for 30 s. Due to the arbitrary distribution of NWs on the SiO${}_{x}$/Si substrate, EBL initally produced arrays of 8 µm markers. The alignment markers’ positions determined the placement of the NWs. They were subsequently contacted by two electrodes separated by a wide range of distances depending on the length of the NWs using the same lithography procedure as previously described. Two probes were used for the electrical characterization through two contacts on the NWs using two different experimental arrangements: “ambient setup” and “vacuum setup”. The former uses a Semiconductor Characterization System with the Keithley Interactive Test Environment; the system is located in a gray room environment at atmospheric conditions in pressure and temperature. The latter is based on an Agilent 4156C Precision Semiconductor Parameter Analyzer; the samples were placed on a substrate holder in a vacuum chamber at a base pressure of 1 × 10^−6^ mbar and at room temperature. The *I*-*V* measurements for both systems were taken in the −20 mV to 20 mV input voltage range in bright light using the power supplies for each system described above. The resistance of two-probe devices was determined by linear fitting of the *I*-*V* curves at room temperature.

## 4. Conclusions

We have demonstrated that the self-assembly of Au NWs inside quasi-circular molds synthesized using the DNA Origami method leads to the successful formation of continuous metal lines. The percentage of NWs exhibiting very low resistance values was increased substantially compared to earlier studies on metalizing six-helix-bundles [41] or rectangular molds [19]. This result indicates that strain induced in the metallic layers during the growth of the NWs may play an essential role during the formation of continuous metal lines. Some of the wires have large initial resistances that dropped by orders of magnitude after initial measurements; these wires behaved very similarly to wires with low initial resistance values in all further measurements. We explain this significant change in resistance with current-induced annealing of the NWs caused by electromigration of the Au atoms on the substrate surface. These results pave the way for the reliable formation of metal NWs and thus the creation of interconnects in nanoelectronic circuits using self-assembly. Such an approach to nanolithography may offer possibilities for energy-saving fabrication of future nanoelectronic components.

## Figures and Tables

**Table 1:** Dimensions of modules as measured cross-section analysis of AFM images.

Module	Width	Length
Purple (P)	34(8) nm	33(8) nm
Blue (B)	36(8) nm	30(8) nm
Green (G)	32(8) nm	32(8) nm
Yellow (Y)	38(8) nm	30(8) nm
Orange (O)	40(8) nm	35(8) nm

**Table 2:** Calculated resistances for the different NWs using a straight line fitting at room temperature and different pressure conditions. *d* and *l* are the diameters and lengths of the wires, respectively, as measured in the analysis of SEM images.

	$R_{\mathrm{RT},\mathrm{amb}}$	$R_{\mathrm{RT},\mathrm{vac}}$	*d*	*l*
A	1500(4) kΩ	365.0(1) Ω	120(10) nm	220(10) nm
B	78.0(4) Ω	56.0(2) Ω	142(10) nm	195(10) nm
C	410.0(2) MΩ	116.0(1) Ω	128(10) nm	183(10) nm
D	5.10(2) GΩ	206.0(4) Ω	116(10) nm	188(10) nm

## Data Availability

Data can be made available on request by the authors.

## Supplementary Materials

The following supporting information can be downloaded at: https://www.mdpi.com/article/10.3390/ijms241713549/s1.
