# Supplementary material for: Metallic Nanowires Self-Assembled in Quasi-Circular Nanomolds Templated by DNA Origami
Source: Int J Mol Sci. 2023 Aug 31;24(17):13549. doi: 10.3390/ijms241713549 (PMC10487803; doi:10.3390/ijms241713549)
Supplement: Supplementary file 1 [file ijms-24-13549-s001.zip › ijms-2516318-supplementary.pdf]

A. Description and sequences of ssDNA in every ¨pool¨ (13 pools)

Pool	No. of staples	Code	Concent.	Description
1	150	NM	0.5 µM	Central section of all modules
2	6	Y	0.5 µM	Staples for yellow NM
3	6	B	0.5 µM	Staples for blue NM
4	4	Au NP1	0.5 µM	Extended staples for attaching Au NP1
5	4	Au NP2	0.5 µM	Extended staples for attaching Au NP2
6	5	YP	0.5 µM	Staples for plugging the Y module
7	6	BP	0.5 µM	Staples for plugging the B module
8	6	BT	0.5 µM	Staples for rejecting the B module
9	6	YT	0.5 µM	Staples for rejecting the Y module
10	6	OP	0.5 µM	Staples for plugging the O module
11	6	O	0.5 µM	Staples for orange NM
12	6	PP	0.5 µM	Staples for plugging the P module
13	6	P	0.5 µM	Staples for purple NM

Table S1. Pools description for assembling the modules and positioning AuNPs.

This table summarizes the staples, or extended staples, which are complementary to the scaffold or to the functionalized Au NPs, in each module, or section of module, per ¨pool¨. The number of complementary staples to the scaffold varies from 170 for the central module to 182 for any other module; see Supp Info B to check the required pools for annealing each module. Extended staples with five thymines were used as plugs if a notch occurs in a module due to an unpaired section. The codes relate to the initial color assigned to each module, but NM, which means the green (**G**) module and the others, are self-explanatory. The staples sequences per pool are described below.

a. *Pool* 1

Name	Sequence
NM_13[49]-10[56]	TGAAATAGCAATAGTTATATA
NM_50[104]-55[113]	CGTTGTAGCTCATGGCCGCTACCACCACACCCGCCGC
NM_59[28]-68[28]	AAATTCGAATTGTACAACCTTCCCGCCA
NM_44[48]-49[55]	TAGCGCGCGCCAGCATTGACAGGCCGCCCAGAACTGAGTAACTGGTAAT
NM_42[104]-41[90]	AGGTGAGCAGTTGGGTTGAAAGGAATTGAGGAACAAATCAAT
NM_23[63]-19[69]	ACGAGAAGCGCGAAGCGACCTGACCAGGCCAGGTG
NM_53[19]-48[12]	ACCCTAAAGCCGGCGATTATTCTTGCCCCCTGCCTATTT
NM_69[28]-76[21]	CCCTGGAACATAAATCATTTCGACCTCCGAATTAC
NM_66[62]-65[69]	TTCTTCTCCAGCTTTCCGGCA
NM_53[32]-19[34]	CCCAAAGCACCTATCAG
NM_20[76]-31[83]	ATACCAAACACCAGCGCCAGGGGGGAGAGGCGGTT
NM_52[69]-51[55]	GGAATAAGAGAGGGCACTTGCCTGAGTAGAAGAACGGATAAG
NM_58[113]-63[104]	CTAGCATGTCAATCATAACGGTATAAAGCCAAATGCA
NM_4[48]-15[55]	TTAAATCGCAAATCAGCTCATCCGCATAATTCTGTATATGTGCTTGCTT
NM_31[84]-20[77]	TGATTTTATGGGCGTAAAGTTTGTACCGTAACACTGTTACTTAGCGATT
NM_11[35]-12[42]	ATTGAGCGCTGCTGGTAAATAGAAACAAGGAAACC
NM_24[90]-30[91]	GGCTTGCCTAAAGAGAATTGGAGTAAAGGCACCAATTTCTGT
NM_64[69]-62[56]	CAAAAACTGCCTGAAGAGGGTAGCTATTCTTCCTGAGTAACA
NM_68[90]-72[91]	GAACGAGAGAGGCTTTTGCAATGTTTAGATGCTGTCCAGACC
NM_9[77]-59[97]	TAGCATTGAAATACCGACCGTACCTAAAAGGCCGGACCGTTCATCGATG
NM_44[90]-48[91]	CATTAAACTTGATATTCACAAAGCGCAGGGATTATATTTTGA
NM_41[91]-45[90]	ATCTGGTGCGGTCAAATTTTAAAAGAAACCACCAGTAGTCTT
NM_0[34]-14[42]	CAATAATTATTAAAAGTATGTGCATGATTAAGACTCCATAACAGTGATT
NM_65[91]-69[90]	ATTAGCAATAAATCCTGTTGGACGCCAGCTGGCGAGAAGTTT
NM_38[41]-0[35]	TTAGAACGAAATAAGATGAATAACAATAAACCAAT
NM_76[34]-72[21]	CCAAAAGTGGTTGGAAACAGGGCTTAAG
NM_18[106]-33[97]	TGACCAACTTTGAAAGACGAGGCGAACCCATTGTCGT
NM_47[42]-43[41]	TGATAATCAGAAAAGATCATAT

NM_17[42]-41[55]	CCACCCTACCCTCAGAGCCACGAGCCGCTTTTCATGAAACGT
NM_62[48]-8[42]	TATAAAAAACGTCAAGAAATTATTATTCATCTAAAGCATCAC
NM_43[91]-54[95]	GGATTCTAATTCGCAGCGCAT
NM_63[84]-65[76]	CCCTCATGGTAAAGATTCAACATTAAATAAAAATTGTTGTACCCGCTTC
NM_58[97]-70[91]	ATGTACCATTCTACATTCTGCCATTCCAACTAAAG
NM_19[19]-50[12]	AAAAACCGTTAAATCGATTAGCGGAGACTCCTCAAGAGA
NM_6[97]-3[90]	AATAAGAATAAACAACGCTCAAAGTTTTGAGGCCACGAGCCAAGCTAAT
NM_75[35]-68[35]	AACAAATCCCCTCGCACTATCTAAATGT
NM_47[16]-56[19]	CACCGGAACCGCAGAGCCGCC
NM_29[98]-76[98]	CTTGCTTTTTCTTAGACAACAGCGGATTCTGACTAAGTAAAA
NM_37[77]-0[77]	GCCCGAATAATACAGGCGACAAATCAATAAAATCGACAAGAA
NM_54[48]-46[35]	TCGTTAGGCCTTGCAGTGCCCGTATAAAAGAGCCGGACAAAA
NM_67[35]-59[48]	ATGACACTCATGCGCTCAGGAAGATCGCATACTTTTTACAAA
NM_42[76]-57[69]	TGCAACAATCCTTTGGAATTATCATCATACAATATTTTGCCTGAGGCAG
NM_23[98]-29[97]	CCTAAAACGTAATGCCACTACCTTTTTCGAGAATATTATCAG
NM_47[84]-44[77]	CTGAAATTCTCTGAATTTACCGATTGGCAAGACAG
NM_20[97]-52[84]	CATCTTTGACCCCCAGCCGGAAGGACAGGGAGGTTGAGGCCA
NM_42[62]-45[48]	GCTGAGAGCCAGCACAATTCGATTATCAGATGATGGCGTAAG
NM_56[113]-43[109]	TAAAGCCAGAATGGAAACAAATACCCTAAAAGCAGAAGATAA
NM_58[48]-63[48]	TAAGCAAATATTTACATTAAATGCGCATATGGGCGGGAGAAG
NM_34[41]-32[28]	GTGGACTGGCGATGATCAAGAGTAATCTCGGATATTTTGCCC
NM_53[49]-34[49]	CAGAGCGTTTTTGGACGTGAACACTATT
NM_21[49]-33[55]	TTGTGTCCTACAACTTCCACA
NM_70[109]-79[113]	TTTAAATATGCATATAACACGCTATTGAAGGGCGATCGGTGC
NM_31[35]-25[41]	TCGTGCCTTAATTGCCAGACA
NM_1[49]-2[56]	CGCCTGACAGGTTTGAAACGCAAAGACAAATCAATCCAGACGCCGACAA
NM_16[69]-1[48]	TCATATGGTTTATTTTGCTTTCCTAATTTACGAGCATGTAGAACGGATT
NM_60[112]-61[104]	TCAATATGATATTCAAGACAGT
NM_37[98]-0[91]	AAAGTTTTAGATTATATCTTTAGAAGATATTCATTTCAACAATAGATAA
NM_2[34]-7[41]	AAGCAAGGCGCCCACGCAATAAATAATAAGAGCAAATATCAGCGCTAAC

NM_22[55]-21[48]	TCATTGTGAATTACCAGTGAAAGATTTGTGATAAA
NM_29[35]-30[42]	GCTAACTCAAAATCGTTGACAAGCTGCAGAGACGG
NM_15[56]-4[49]	CTGTAAACAAAGTTGAAGGCTGAGGTTTTGAAGCC
NM_77[70]-72[70]	AAACCAAAATACTGAGAGCTTACAGGTC
NM_70[41]-67[48]	GCATTTCGTGACTCGGTTTTCCCAAGCTTTCTCAGTGTGAATCTGTTTA
NM_19[70]-34[84]	TATCACCTTCCAGTTAGCCCGAGATAGGGTCACCAAGAGCTG
NM_65[28]-69[27]	TATCGGCCACGACTGCCAGTGCCAGTCACGACGTTACGCTCG
NM_67[84]-63[83]	GGCATCACCGGTTGATAATCAGAGCAAATAAATCGTTTAGAA
NM_72[69]-75[69]	AGGATTATCGAATTGTTTACAGGTAGAAAGAAAACGAGAATG
NM_0[90]-17[109]	GTCCTGACGCAGAGGCGAATTGATGAAAGAAAACAAAATTAATTACA
NM_2[76]-12[84]	GAGAATATATTCTAAGCAGATACCGAAGCCCTTTTTAAGAGTTGAGAAG
NM_23[49]-24[56]	TAAGGCTCACCAGTTTAATGAATCGGCCAATAATTGTAGCAAGCGGGAT
NM_15[63]-17[69]	TCGTCGCGTACATACCACGGA
NM_3[91]-4[98]	GCAGAACATTTGAAAATCCTTGAAAACATTAGATTCATGTAAGAATCGC
NM_77[98]-65[106]	AAGAAGTATTCCCATAATAGTACATCCAAAATTAAGC
NM_60[48]-64[28]	GATCTTTTGTTAAACAAAAATCCGTGGGAACAAACGGTGTAGCGTAACC
NM_41[21]-45[27]	GAGCCATTTGGGAACAAAATCTTATACTATCAATATAATCCTTTTGCCA
NM_7[63]-1[69]	ATTCTTACTTGCGGTATCCGGTAAAGTAACGACAAATCCCATGAATACC
NM_44[76]-52[70]	AATCAAGTTTTGAACATTCTGGCAAGTGTTTGACGGGCCGATATAGCCC
NM_51[56]-48[63]	TGCCGTCATCAAGTGGAGCTAAACAGGAAGCACGTACAATATGGGGTCA
NM_48[76]-50[84]	TTTTAACTACCGCCATAACATTTGATATAATAAAAGAGTCTGTTCTTTG
NM_57[28]-49[34]	ACCACCACACCCTCCTCCCTCCAGTTAAGAAACAT
NM_4[83]-8[77]	AGCGAACCTCCCGACCAGTATCGGCTTAAAAGAACGCGAGAAGTGCCTT
NM_72[90]-26[91]	GGAAGCACTTCAAAAAAGATTGTTGCGCCGACAATAACAGCT
NM_38[76]-43[69]	TTTGAGGATTTAGAGTATTAAGTGCCACCAGCACC
NM_34[34]-20[28]	CCAACGTGGTGGTTCCGAAATACGCTGGTCTCGCCTATCAATTACCCAA
NM_2[55]-12[49]	AAGGTAAAGATATAACCAGAA
NM_52[111]-53[111]	AGAAGTGTTTTTATCGGTACGCCAGAAT
NM_28[55]-27[62]	TTTTCACTCCAAAAAAAAGGCTCACAATGGAACAA
NM_31[91]-24[91]	GCTAAACAACTTTCGCGGAGTATGAGGATATTCGGTCGCTGA

NM_67[63]-65[55]	TGGGGCGCCCAAAAACAGGAAAGGTCATATTATGACCCTGTAACTCCAG
NM_77[49]-78[56]	TCGTTTAATATTCAGGTCATTTTTGCGGAGGGGCCTGCGGCCGATTAAG
NM_61[84]-6[77]	GTGAGAATTTAATGAACTTTTACCTTTTTAACCTCAAAGCCACCGGAAT
NM_50[83]-46[84]	ATTAGTAAGCCATTTGGTTGCTAGCGGTCACGCTGAGTAATA
NM_35[49]-20[49]	ATCCCTTATAAATCGTACAAAGAAATCCACAAAGTACAACGG
NM_58[76]-77[69]	GAAAAGCCGAGCTGAGTTTGACCATTAGACGATAA
NM_47[63]-36[77]	ATACAGGAGTGTACTGGTAGTAAAGGGATGGCTATAAGGAGC
NM_75[28]-71[41]	GATACATGGAATACCCTCGATAAAGACGTTTGAGTTGTAATT
NM_79[56]-69[69]	CAGGGTGGACGCCATTCGCCAATGTGCTGCAAGGCCTGCCAT
NM_24[41]-26[28]	GCGAAAGTCAGGACAAAATCTAAGCATA
NM_48[62]-54[49]	GTGCCTTTTTGATGGAGATAGAACCCTTGGAGCGGGCTTTCC
NM_37[21]-57[27]	GTTTGGAACCAGTACATTAGCAAGGCCGCGGCATTTTCGGTCCAGAACC
NM_11[49]-6[63]	ATGCAAATCCAATCCATAAAAGTTTAACGTCAAAAACTAGAA
NM_43[70]-39[76]	GTAATCAGTAGCTACACCGCCAACCCTCACCGATTGAGGGAGACAAAAG
NM_66[55]-77[48]	AAGTGGTGAGAAGCTTGGGTAACGCCAGTATGATATGGTCAAATAACCC
NM_42[41]-38[21]	AAGCCAGTTAGAAAATTAAAGGTGAATTTAAAACACTACCATATCAAAA
NM_41[56]-38[42]	CTTGCTGAAATATTGACGGAAGCGTAGAGACTTTACAAAGGG
NM_33[28]-34[42]	AGTTGCAGCAAGCGGTTAGCAGCCTGCCCGGCAAAAAAGAAC
NM_29[63]-35[69]	TCCAAAATGCGAATAACGCGCGTGGTTTCTCATAGGTCACCAAAAAGAA
NM_57[70]-43[62]	GTCAGACGTTCCAGTAAGCGTCATACATGGAGGTTTTAGCGTTCGATAG
NM_34[83]-33[76]	AGTGTTGGTACTCAATGAACGGTGTACAGCTCCATGAGTTTCTTAGCGT
NM_65[12]-60[19]	GAGGGGACGACGACAGGTGCATCTTTGTTAATTTTTTAA
NM_63[21]-13[27]	TCACGTTGGCGGATAATAGGAAACTGAAAAAGTCAACCCACATAAGCCC
NM_75[70]-70[77]	ACCATAAAGTCATGCGTAACCCGAAAGAAACTCCAAATTGCTTCCGTCG
NM_65[77]-61[83]	TGGTGAGGCAAAGAATAAAGCCAAGAGATAGCTGATAAATTACAAAAGG
NM_12[41]-11[34]	GAGGAAAATAGCAAAAGATTAGTTGCTATTACCAAAGAGATAGAGGGTA
NM_62[55]-8[63]	ACCCGTCCCTTTATTTCAACGCAAGGATGTGAGCGTAGCCAGAGAATAA
NM_34[69]-18[49]	TTGGAACAAGAGTCCCATCACCGCATAGGCTGGCT
NM_73[42]-25[55]	GAGGATCTCAGTTGTAAAACGCTCAGCAGCATCGGAACGAGG
NM_77[77]-66[63]	AATAGCGTAGATTTAAAAGGTCACCGGAAACCAGGCAAAGTG

NM_4[97]-9[104]	CATATTTACAGTAGAGAGACTTCAAATATATTTTATCTTCTGGTGATAA
NM_45[35]-43[48]	ATAATAAGCAATTCTCTGAATAATGGAAGCAAATGCACCAAT
NM_39[91]-43[90]	TCTAAAAGAGCCGTCAATAGACGTTATTGTATTAACCGAACG
NM_0[76]-5[83]	AAATAATTAAACAAGGAAACATATTAATTAATTTTAAAAGTAAGAACGC
NM_37[56]-41[76]	ACAACTCAGTATTATTCCAGCGCCAAAGGGAAGGTAACCTCAAATATCA
NM_69[70]-66[84]	CTGTAAGCAACTTGAAGGGGGTTCAGGCTGCGCAAATACAGG
NM_6[41]-60[28]	CCAGAGCCTAATTTTCCCAATGAGAATTACGCCATTCAGCTC
NM_68[55]-75[48]	TCGCAAACCGACAGTTGAATCGGCTGACGATAAGATTGAATCGCTTTAA
NM_7[56]-60[49]	TATACAAAAAGCCTGTTTAGTATTTTTTACAGGGAGTCTGGCTTTGAGA
NM_26[90]-28[84]	TGATACCTATCGGTGAAAGGA
NM_1[28]-2[35]	CGGGAGAATACAGTTAAAGGTGGCAACAATTACGCCCAAGTACGAGAAC
NM_28[83]-26[63]	ACAACTATTGAGGAAGGGAGTTAAAGGCTAGCTGTAAATTGTTATCCGC
NM_31[16]-30[16]	TTTCCAGTCGGGCCCTTCACCGCC
NM_50[34]-53[48]	AGAGGCTGGGTTTTGCTCAGTGTGCCGTCCGATTTAGAGAAT
NM_1[70]-39[90]	AAGTTACAGAAAATATAAGTTTATTTTGTCATCAACAAACACTTGGTTA
NM_30[41]-29[34]	GCAACAGGCTCATTCTTATGCGATTTTACATTATACGTTGCGTGAGTGA
NM_18[48]-50[35]	GACCTTCGCCCACTGGTCGAGACCAGGCTCAAACTATTATTA
NM_54[94]-35[104]	GGCGACAGGAAAATCAGTTAGTACCCACCCTCCCCTCATTTTCAGG
NM_15[84]-2[77]	CCCTTAGTTACCTTTTTTAATCATGTTCGTAATAA
NM_70[90]-75[90]	TACGGTGGAATATAACTGGATATCAGGT
NM_29[70]-76[77]	GGAGCCTTTAATTGGAGTGTGTTCCTTAAAGAGGAATCAAAAAGCGTCC
NM_24[55]-74[35]	CGTCACCAACTAACTCCACACAACATACGAGCCGGACGTTAAAGATTTA
NM_75[49]-72[42]	ACAGTTCAGATTCACCCGGGTACCGAGCGAGAGTACCTTTAATTGCTCC
NM_48[104]-36[98]	TACCTACTTACATTCCAGTCACGAACTGGCGGAAC
NM_49[35]-46[42]	GAAAGCGCTTGACGGGAAGAAAGCGAAACTGACCT
NM_8[41]-10[49]	TAGACGGCCAAATAAGAAACGATTCTTTGAGCGCATATGCGTACTATAT
NM_52[83]-48[77]	CCGAGGTTAAAGGGATTTTAGCGTACTAGCAACAGCGCTCAATCATAAG
NM_33[77]-28[70]	AACGATCTATTGGGAACGAGTGCTTGAGATGGTTTAGAGGCTAAGGAAT
NM_27[63]-22[56]	CATTACACGCTTTTCGGCTACAATTTCAACTTTAA
NM_6[76]-59[76]	CATAATTATGAAAATACAGAGCTTTCATATGCCGGGAGTCTG

NM_43[49]-36[56]	GAAACCACAGACTGAATACGTGGCACAGATTCCTG
NM_70[76]-68[56]	GTGGGCACGAATATATGGCTTCGGAATCGTCATAACCAGACGATACATT

b. ***Pool*** **2**

**Name**	**Sequence**
Y_17[16]-40[6]	AAA ATA CAT ACA AAC AGT ATT GCA CGA TCA CCG TCA CCC AAC ACA AAT
Y_26[27]-28[6]	AAG TGT ATG CCT AAC TCA CTG CCC GCT TTC AAA CAG
Y_33[4]-34[21]	CTT TCC AGA CGT TGG CCC TGA GAG CAG CAG GGT TTG ATC AAA GGG
Y_3[3]-0[12]	GCG CCT GTT TCA TTC CAA GAA CGG GCG GCT GTC TTT CCT TA
Y_7[3]-5[27]	GGC TTA ATT AAT TTT ATC CTG AAT CTT TTG CAC ATT ACC
Y_9[14]-6[3]	AGC CAT ATT ATT TAG CCA GTT ACA AAA TAA AAG GCG TTA

c. ***Pool*** **3**

**Name**	**Sequence**
B_26[109]-29[119]	AAA ATA CAT ACA AAC AGT ATT GCA CGA TCA CCG TCA CCC AAC ACA AAT
B_32[119]-32[98]	AAG TGT ATG CCT AAC TCA CTG CCC GCT TTC AAA CAG
B_49[91]-51[115]	CTT TCC AGA CGT TGG CCC TGA GAG CAG CAG GGT TTG ATC AAA GGG
B_2[121]-13[97]	GCG CCT GTT TCA TTC CAA GAA CGG GCG GCT GTC TTT CCT TA
B_75[91]-73[121]	GGC TTA ATT AAT TTT ATC CTG AAT CTT TTG CAC ATT ACC
NW_B_62[115]-64[91]	AGC CAT ATT ATT TAG CCA GTT ACA AAA TAA AAG GCG TTA

d. ***Pool*** **4**

Name	Sequence
NW_S1_49[56]-46[119]	ATC CAG AAT AAC GTG CGC TAG GGC GCT GGC CAA CAT TTT TAA TAA TAA TAA TAA
NW_S1_33[56]-23[137]	GAC AGC CTT CTT TTT GCC CTG TTT TTA ATA ATA ATA ATA A
NW_S1_59[49]-67[137]	GGC TAT CGA TTG TAG CTA TAT TTT CAT TTT TTT AAT AAT AAT AAT AA
NW_S1_11[63]-12[119]	GCA AGA CGG TTG GGC TAT CTT AGC CGA ATT TTT AAT AAT AAT AAT AA

e. ***Pool*** **5**

Name	Sequence
NW_S2_45[119]-53[31]	AAA AAA AAA AAA AAA TTT TTC TTT TCT CAC CGG AAG GAA GGG GAA AGG GAG
NW_S2_68[137]-77[34]	AAA AAA AAA AAA AAA TTT TAA ATA ACC ATA GTA AGA GCA A
NW_S2_15[119]-14[21]	AAA AAA AAA AAA AAA TTT TAT AAC GGA GAA CTG TAG CAA A
NW_S2_22[137]-31[34]	AAA AAA AAA AAA AAA TTT TCA AAG CTC TGA TTG AAA CCT G

f. ***Pool*** **6**

Name	Sequence
TY_40[114]_41[114]	TTT TTA AAT CTT GGC TTT TT
TY_3[98]_3[111]	ACG CGC CTG TTT TT
TY_6[111]_7[111]	TTT TTA TAA GGC GTA GGG CTT AAT TTT T
TY_28[114]_31[114]	TTT TTA GTT TTC AAC TTT TT
TY_33[98]_33[114]	GTC TTT CCA GAC TTT TT

g. *Pool* 7

Name	Sequence
TB_73[11]_73[27]	TTT TTT ACG TGG TGC TT
TB_62[20]_62[7]	TGA CCG TAA TTT TT
TB_35[11]_32[11]	TTT TTG AAA ACA GGC TTT TT
TB_29[11]_26[11]	TTT TTA GCC TTG TAA TTT TT
TB_51[7]_51[20]	TTT TTG AAG GAT TA
TB_2[27]_2[7]	AGC CGT TTT TAT TTT CTT TTT

h. *Pool* 8

Name	Sequence
BT_75[91]_73[114]	CTT TAC CGC ATC AAT ATC GCG TTT TAA TTC GAG TTT TT
BT_2[111]_13[97]	TTT TTT AGG CAG AGG CAT TTT ACG CCA AAA GAC GCC AAT AGT
BT_26[109]_29[114]	GTG AAT CGA GTT TTT
BT_62[111]_64[91]	TTT TTG CCT GAG TAA TGT GTA ATA TTT TTC AGA GC
BT_49[91]_51[111]	GAA AAA CGC AAT ACT CCA TCA CGC AAA TTA TTT TT
BT_32[114]_32[98]	TTT TTC AAG CCC AAT AG

i. *Pool* 9

Name	Sequence
YT_17[16]_40[11]	AAA ATA CAT ACA AAC AGT ATT GCA CGA TCA CCG TCA CCT TTT T
YT_33[11]_34[21]	TTT TTT GGC CCT GAG AGC AGC AGG GTT TGA TCA AAG GG
YT_9[14]_6[7]	AGC CAT ATT ATT TAG CCA GTT ACA AAA TAA TTT TT
YT_26[27]_28[11]	AAG TGT ATG CCT AAC TCA CTG CCC GCT TTT T
YT_7[7]_5[27]	TTT TTA ATT TTA TCC TGA ATC TTT TGC ACA TTA CC
YT_3[7]_0[12]	TTT TTT CAT TCC AAG AAC GGG CGG CTG TCT TTC CTT A

j. *Pool* 10

Name	Sequence
TO_46[114]_47[114]	TTT TTA TTC AGG CAG TTT TT
TO_42[114]_42[105]	TTT TTA ACA G
TO_8[114]_11[114]	TTT TTT TTC AGT TAA TTT TT
TO_12[114]_15[114]	TTT TTA TAG CTA GCG TTT TT
TO_66[114]_67[114]	TTT TTC ATT AAG TAG TTT TT
TO_74[118]_74[105]	TTT TTG AAG CAA A

k. *Pool* 11

Name	Sequence
O_75[19]_74[11]	ACT AAT GCA CAC ATT CAG AAG CAA A
O_46[27]_46[6]	AAC CAG AGC CAC ATT CAG GCA G
O_43[16]_42[11]	ATT ACG CAC CAA CAG
O_67[16]_66[6]	GTG CTG AAT TGT TAA GTG TCC TTA GAT TAA GTA G
O_11[16]_8[6]	TGA ACC ACC CTT TCA GTT AA
O_15[16]_12[6]	CCA AAA ATA CAT AGC TAG CG

l. *Pool* 12

Name	Sequence
TP_25[18]_22[18]	TTT TTT GGC TAG AAC TTT TT
TP_13[11]_10[11]	TTT TTT GAG TAG AAT TTT TT
TP_72[27]_72[11]	GCT TAA GCT ACG TTT TT
TP_23[18]_20[18]	TTT TTC GTA AAT CAA TTT TT
TP_27[18]_24[18]	TTT TTG AAG AGT TGG TTT TT
TP_14[20]_147[11]	CGT AGT TTT T

m. *Pool* 13

Name	Sequence
P_20[126]_20[98]	CGT AAA TCA AAA GAA TAC ACT AAA ACA CT
P_14[114]_14[98]	CGT AGT TTA ACA ATT TC
P_10[114]_13[109]	TGA GTA GAA TAT CAT AGG TCT GGA ATT TAT CAA A
P_24[126]_25[116]	GAA GAG TTG GGC ATA ACC GAT AAG TTT CCA TTA A
P_72[121]_71[109]	GCT TAA GCT ACG CTT CAA AGC GAA AGC TCA ACA TGT
P_22[126]_23[116]	TGG CTA GAA CAC GGG TAA AAT ACG AAA GAG GCA A

B. Volumes of buffer, scaffold and necessary pools for annealing every module

Sample	Initial Concentration	Final Concentration	Volume
Scaffold	0.1 µM	0.01 µM	10 µl
Pool 1	0.5 µM	0.05 µM	10 µl
Pool 4	0.5 µM	0.05 µM	10 µl
Pool 5	0.5 µM	0.05 µM	10 µl
Pool 8	0.5 µM	0.05 µM	10 µl
Pool 9	0.5 µM	0.05 µM	10 µl
Pool 12	0.5 µM	0.1 µM	20 µl
Pool 13	0.5 µM	0.05 µM	10 µl
10 × TAE Mg^{2+}		1 × TAE Mg^{2+}	10 µl
		**TOTAL**	100 µl

Table S2. Contents for annealing the purple module.

Sample	Initial Concentration	Final Concentration	Volume
Scaffold	0.1 µM	0.01 µM	10 µl
Pool 1	0.5 µM	0.05 µM	10 µl

Pool 3	0.5 µM	0.05 µM	10 µl
Pool 4	0.5 µM	0.05 µM	10 µl
Pool 5	0.5 µM	0.05 µM	10 µl
Pool 7	0.5 µM	0.1 µM	20 µl
Pool 9	0.5 µM	0.05 µM	10 µl
10 × TAE Mg^{2+}		1 × TAE Mg^{2+}	10 µl
DDi H_2O			10 µl
		**TOTAL**	100 µl

Table S3. Contents for annealing the blue module.

Sample	Initial Concentration	Final Concentration	Volume
Scaffold	0.1 µM	0.01 µM	10 µl
Pool 1	0.5 µM	0.05 µM	10 µl
Pool 4	0.5 µM	0.05 µM	10 µl
Pool 5	0.5 µM	0.05 µM	10 µl
Pool 10	0.5 µM	0.1 µM	20 µl
Pool 12	0.5 µM	0.1 µM	20 µl
10 × TAE Mg^{2+} (200 mM)		1 × TAE Mg^{2+} (20 mM)	10 µl
DDi H_2O			10 µl
		**TOTAL**	100 µl

Table S4. Contents for annealing the green module.

Sample	Initial Concentration	Final Concentration	Volume
Scaffold	0.1 µM	0.01 µM	10 µl
Pool 1	0.5 µM	0.05 µM	10 µl
Pool 2	0.5 µM	0.05 µM	10 µl
Pool 4	0.5 µM	0.05 µM	10 µl
Pool 5	0.5 µM	0.05 µM	10 µl
Pool 6	0.5 µM	0.1 µM	20 µl
Pool 8	0.5 µM	0.1 µM	20 µl
10 × TAE Mg^{2+} (200 mM)		1 × TAE Mg^{2+} (20 mM)	10 µl
		**TOTAL**	100 µl

Table S5. Contents for annealing the yellow module.

Muestra	[inicial]	[final]	Volumen
Scaffold	0.1 µM	0.01 µM	10 µl
Pool 1	0.5 µM	0.05 µM	10 µl
Pool 4	0.5 µM	0.05 µM	10 µl
Pool 5	0.5 µM	0.05 µM	10 µl
Pool 8	0.5 µM	0.1 µM	20 µl
Pool 9	0.5 µM	0.1 µM	20 µl
Pool 11	0.5 µM	0.05 µM	10 µl
10 × TAE Mg^{2+} (200 mM)		1 × TAE Mg^{2+} (20 mM)	10 µl
		**TOTAL**	100 µl

Table S6. Contents for annealing the orange module.

C. Gel electrophoresis of one module

Gel electrophoresis is a common tool in many labs for resolving the presence of nucleic acids. Discrete bands are observed in a gel due to the charge of macromolecules when an electric field is applied. The distance that a band moves in a gel basically depends on the mass of the macromolecules, i.e., the length of the nucleic acid. The smaller nuclear acids travel farther compared to the larger ones. The intensity of a band is proportional to its content of nucleic acid when illuminated by a lamp; usually in the UV range. An estimate of DNA size in a sample is obtained by comparing the band position from a standard curve that is based on a set of macromolecules of known molecular mass, number of DNA base pairs, also known as size markers. The size markers are included in the same gel as the sample because the extent of mobility of DNA strands varies for every electrophoretic run. The assessment of the formation of modules, and their possible combinations, was done by observing electrophoretic band shift in agarose gels for every module under different annealing conditions. Figure C.1 shows a typical electrophoretic mobility study for the synthesis of the ¨blue¨ module. The lanes labeled as ¨ladder¨ and scaffold correspond to the size markers and the modified phage plasmid having a total length of 7249 + 311 nt (3780 bp), respectively. The other lanes shown in the gel are related to the blue¨ module synthesis ¨ using different experimental conditions, see the tables in the Supp Info A and B. The bright discrete bands framed with a dashed rectangle, having a length size of approximately 8000 bp, provide confirmation of the module realization, and verified by AFM. However, smear of faint stained material and narrow dim bands are also observed on bottom and top, respectively, of the image in the figure shown below. Comparing with size markers in the ¨ladder¨, the former one is due to the leftover of unbound staples after annealing having sizes in the order of tens of bases, the latter one is most probably because of the formation of undesired secondary structures with sizes above 16000 bp. The electrophoresis results for the other four modules (purple, green, yellow and orange) are similar to the one shown here.

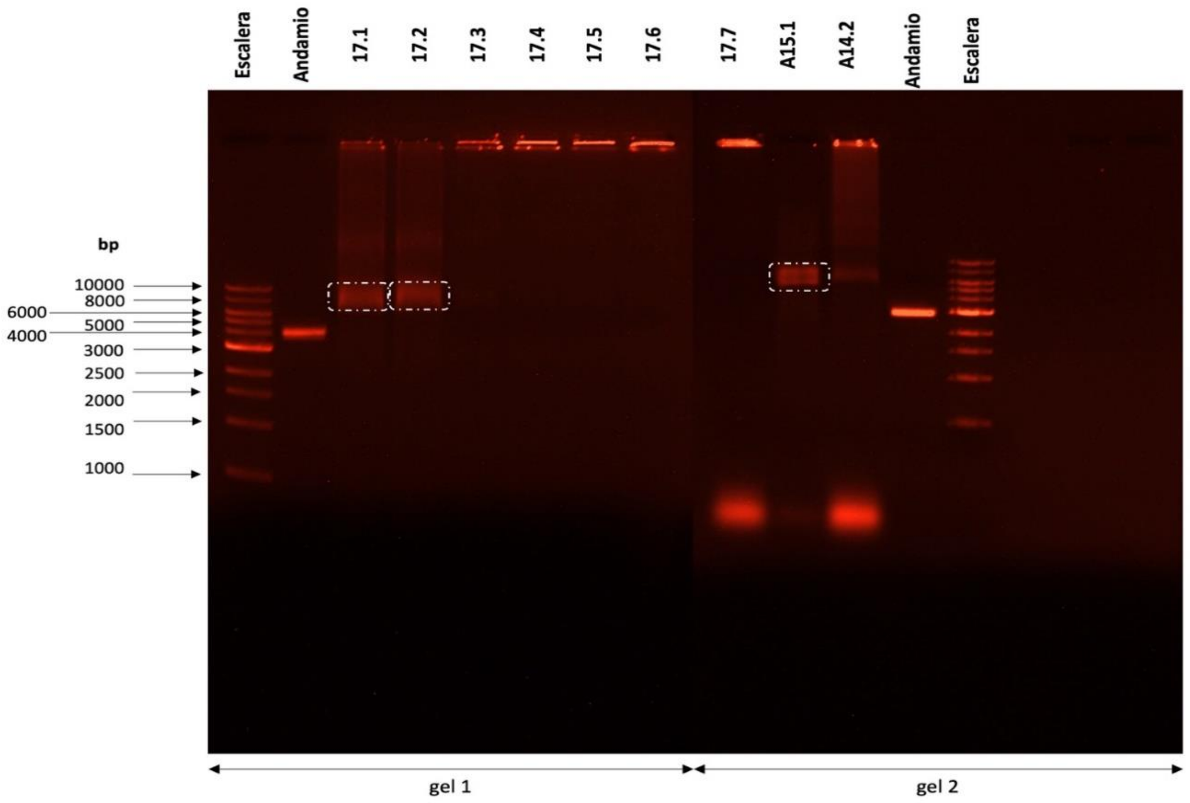

Figure S1. Agarose gel electrophoresis image of the ¨blue¨ module, ¨bp¨ means DNA base pairs.

D. Variations in Mg^{2+} concentration during synthesis studied by AFM

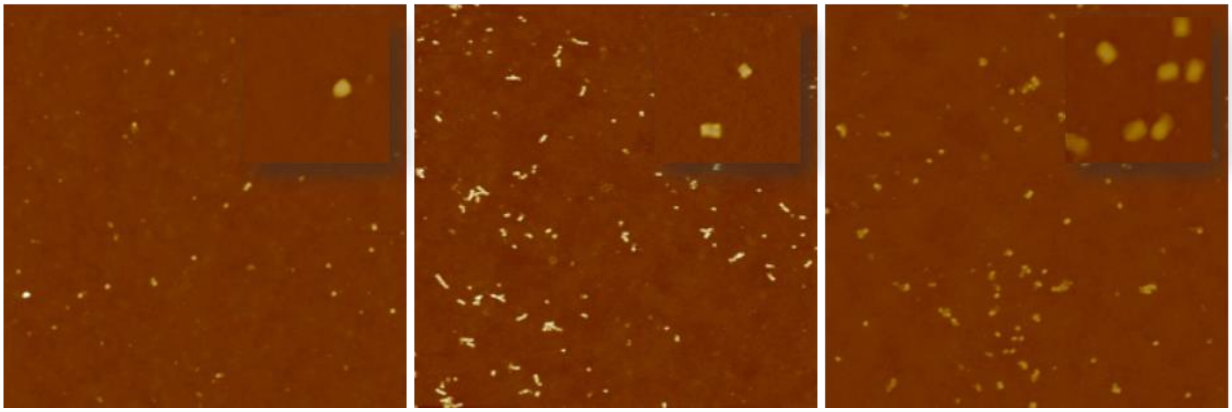

Figure S2. 5 × 5 µm and 1 × 1 µm, upper right corner inset, AFM images showing the purple module deposited on SiO_x/Si substrates at Mg^{2+} concentrations of a) 125 mM, b) 200 mM y c) 350 mM.

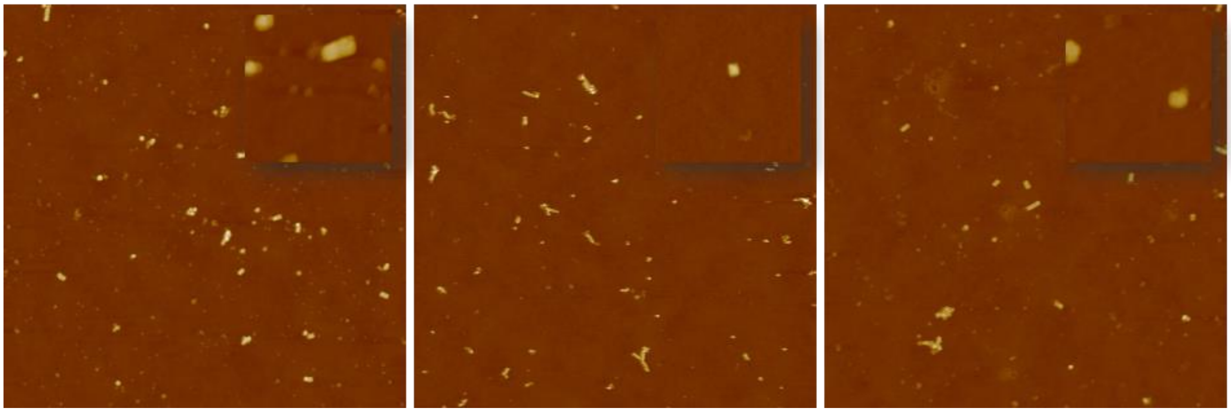

Figure S3. 5 × 5 µm and 1 × 1 µm, upper right corner inset, AFM images showing the blue module deposited on SiO_x/Si substrates at Mg^{2+} concentrations of a) 125 mM, b) 200 mM y c) 350 mM.

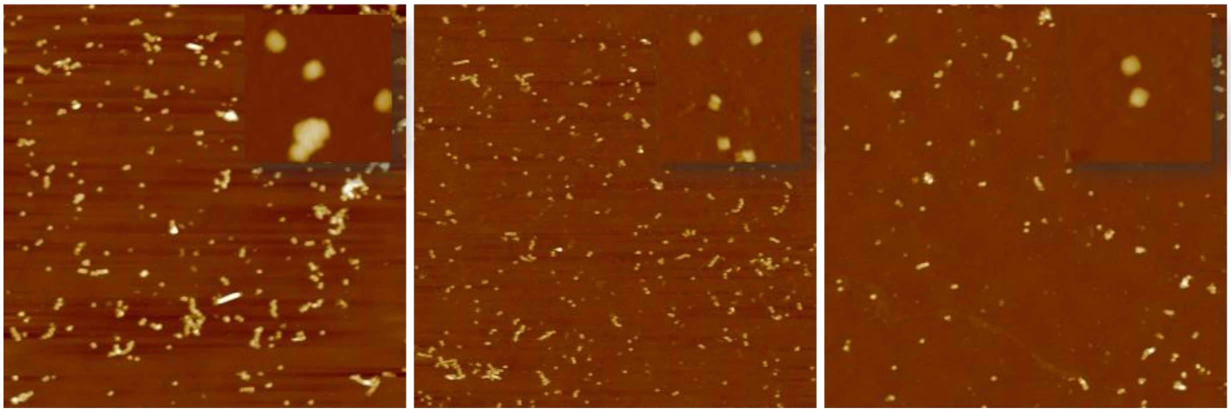

Figure S4. 5 × 5 µm and 1 × 1 µm, upper right corner inset, AFM images showing the green module deposited on SiO_x/Si substrates at Mg^{2+} concentrations of a) 125 mM, b) 200 mM y c) 350 mM.

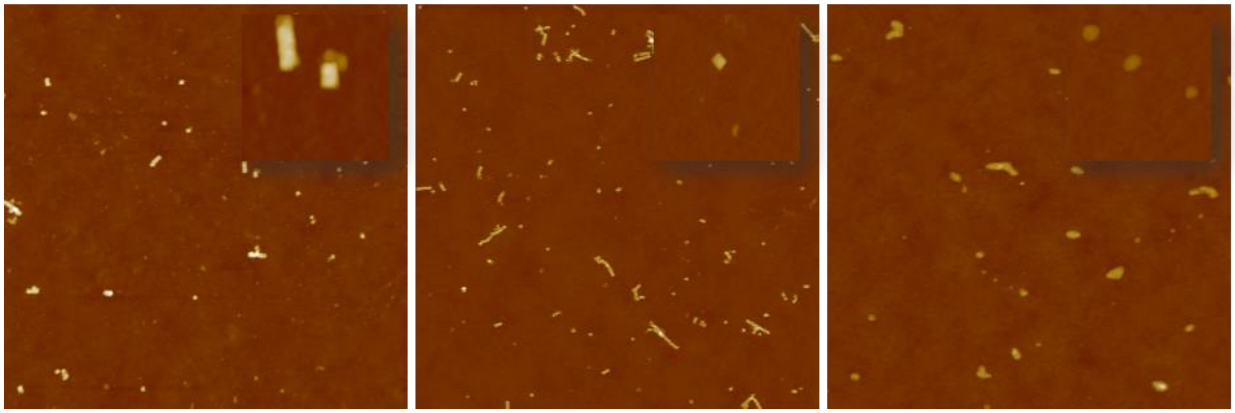

Figure S5. 5 × 5 µm and 1 × 1 µm, upper right corner inset, AFM images showing the yellow module deposited on SiO_x/Si substrates at Mg^{2+} concentrations of a) 125 mM, b) 200 mM y c) 350 mM.

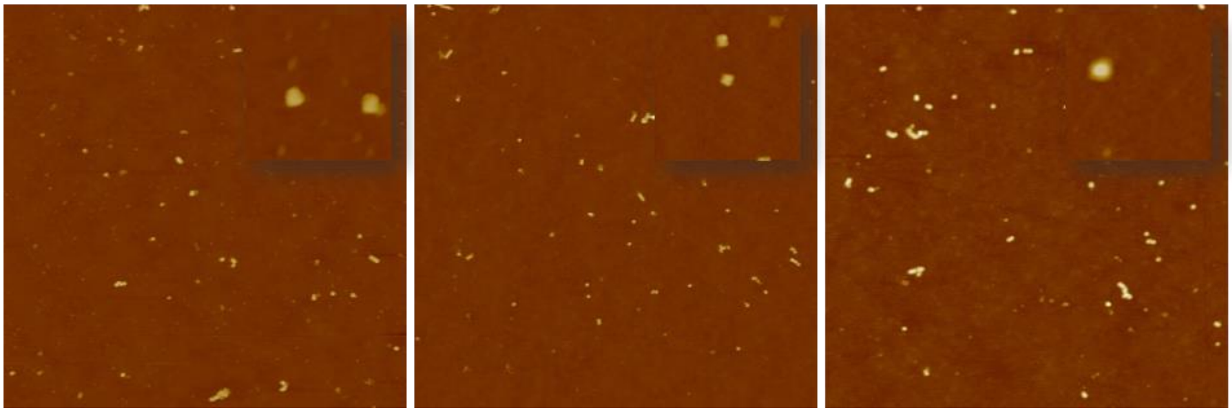

Figure S6. 5 × 5 µm and 1 × 1 µm, upper right corner inset, AFM images showing the orange module deposited on SiO_x/Si substrates at Mg^{2+} concentrations of a) 125 mM, b) 200 mM y c) 350 mM.

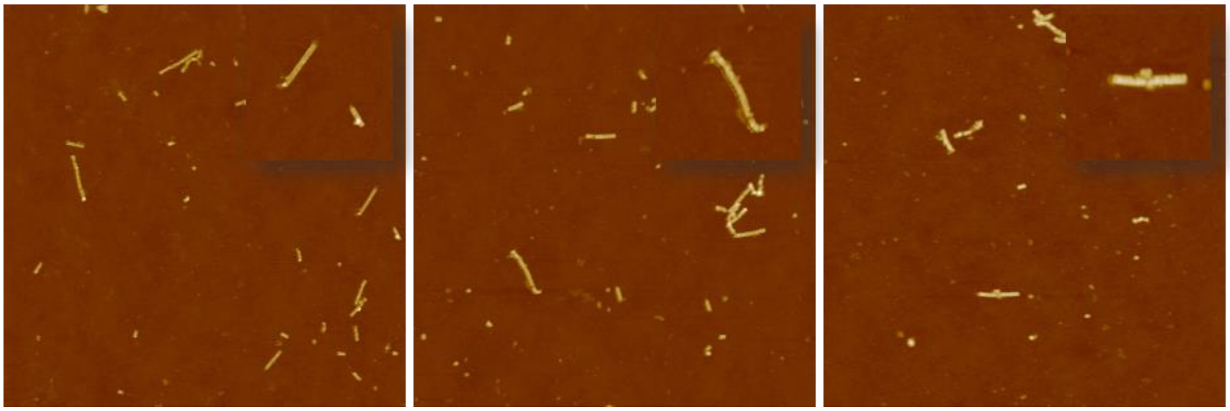

Figure S7. 5 × 5 µm and 1 × 1 µm, upper right corner inset, AFM images showing the basic nanostructure shaped by all five modules deposited on SiO_x/Si substrates at Mg^{2+} concentración of a) 125 mM b) 200 mM y c) 350 mM.

E. AFM images for all modules

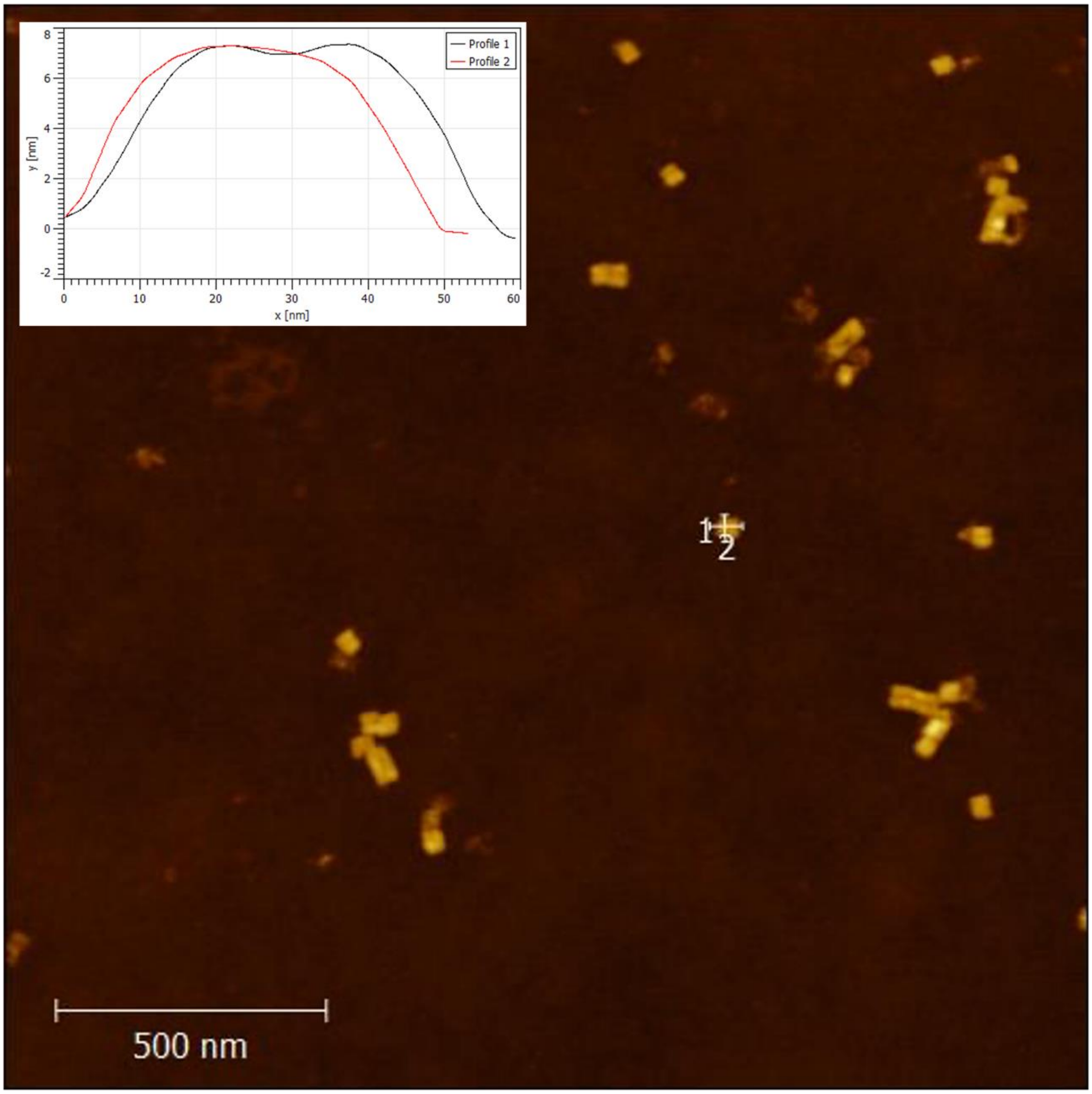

Figure S8. AFM image of the ¨purple¨ module deposited on SiO_x/Si, 2.5 × 2.5 µm size.

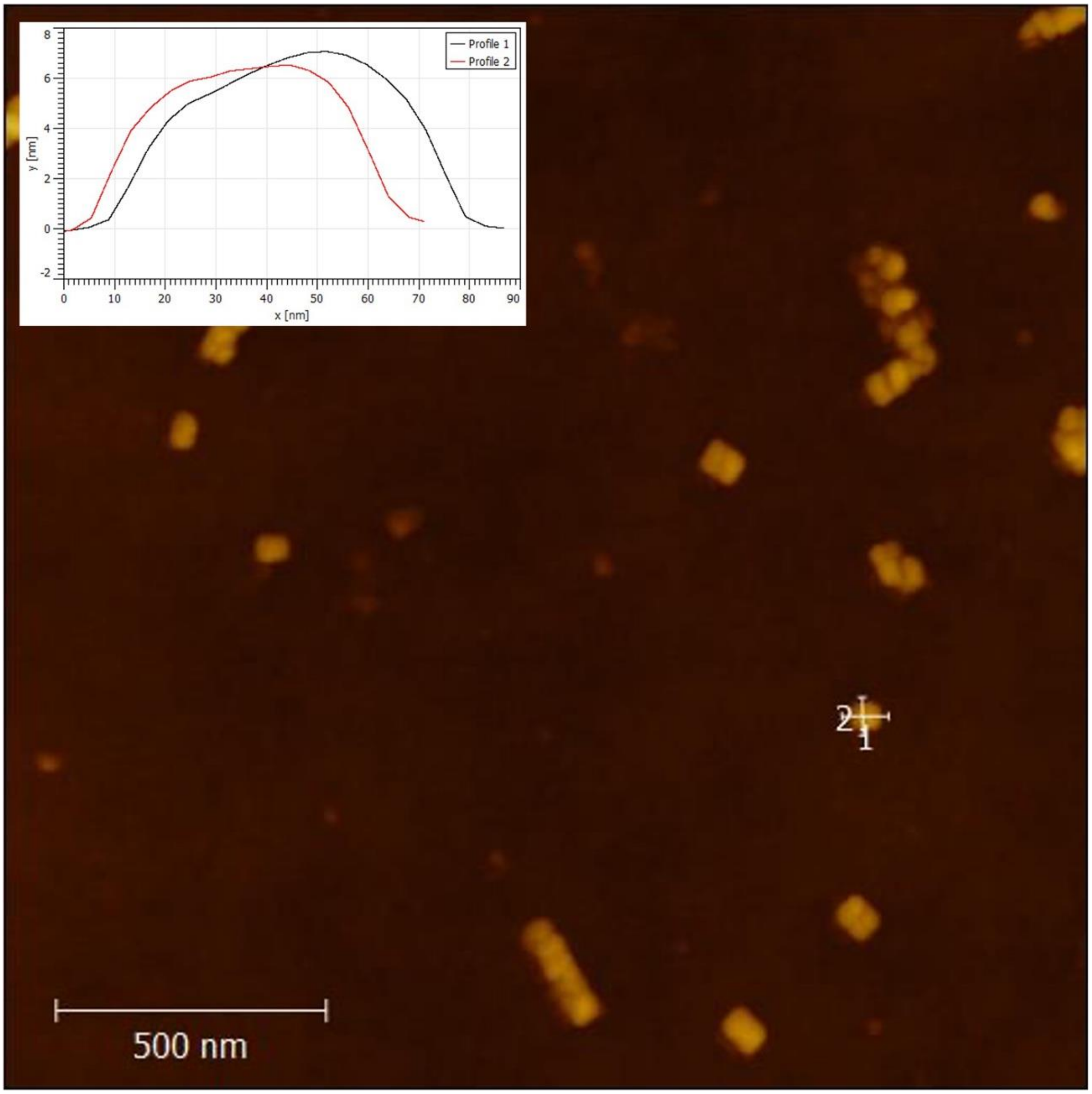

Figure S9. AFM image of the ¨blue¨ module deposited on SiO_x/Si, 2.5 × 2.5 µm size.

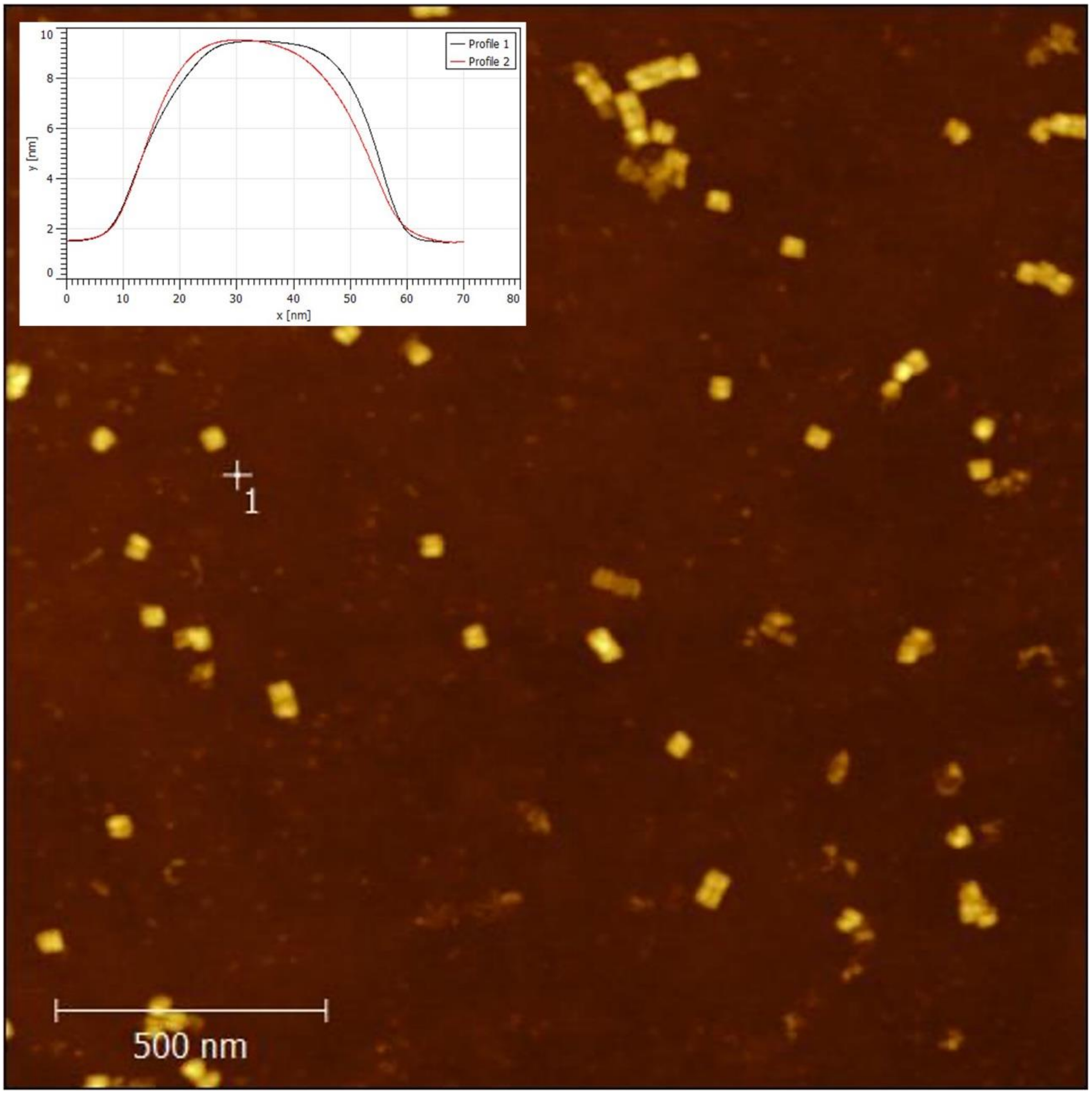

Figure S10. AFM image of the ¨green¨ module deposited on SiO_x/Si, 2.5 × 2.5 µm size.

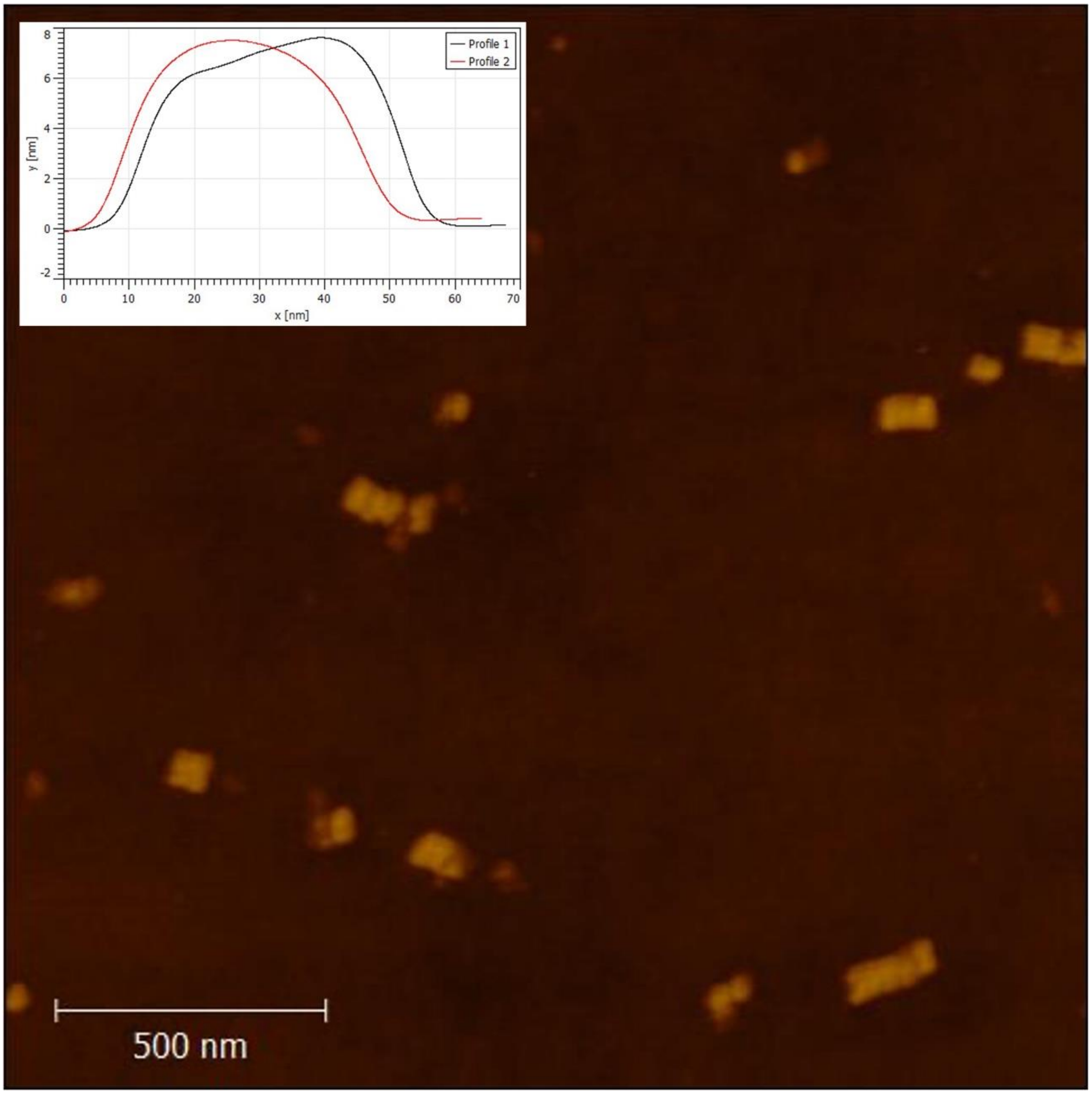

Figure S11. AFM image of the ¨yellow¨ module deposited on SiO_x/Si, 2.5 × 2.5 µm size..

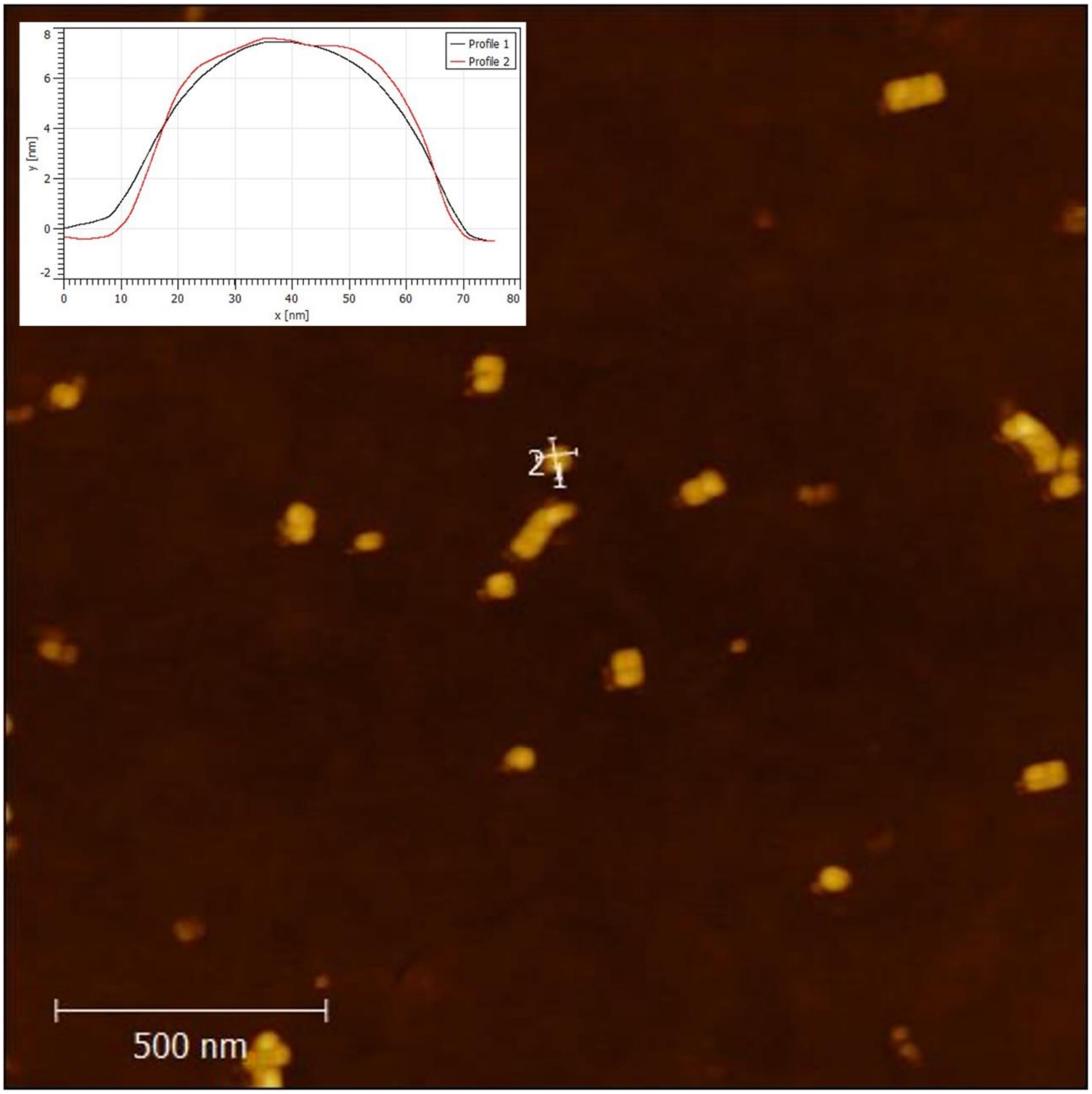

Figure S12. AFM image of the ¨orange¨ module deposited on SiO_x/Si, 2.5 × 2.5 µm size.

F. SEM images of connected nanowires

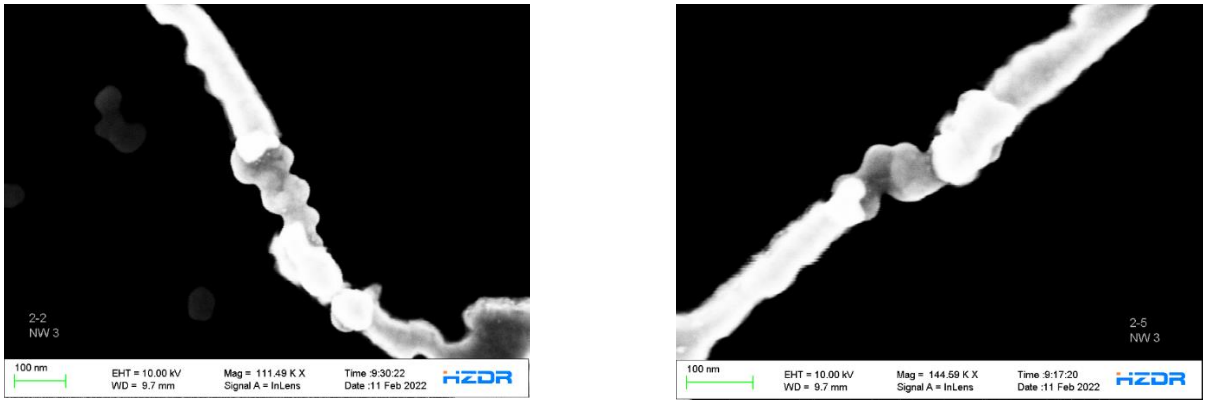

Figure S13 a). SEM micrographs of the interconnections at nanowires A (left) and B(right).

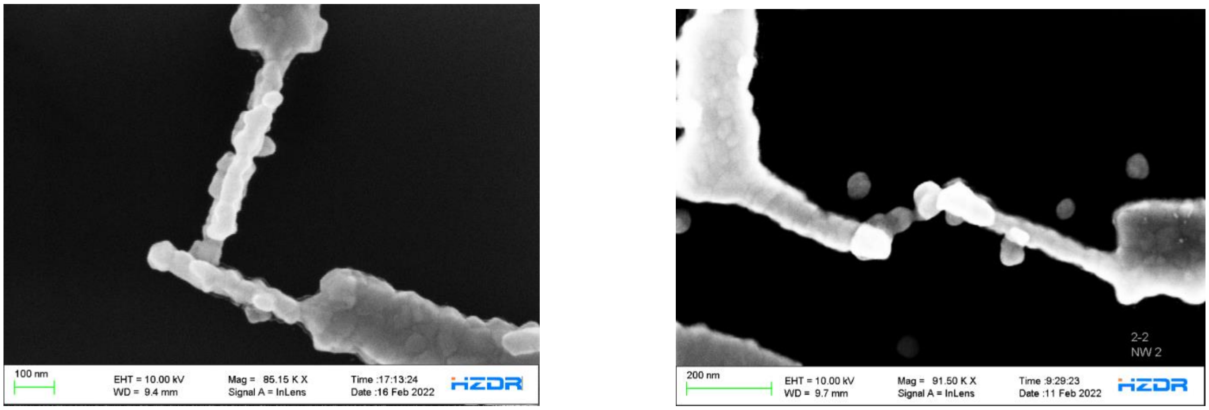

Figure S13 b). SEM micrographs of the interconnections at nanowires C (left) and D (right).
